# Assessment of the Microbiological Potential and Spectroscopic Properties of New Imino-1,3,4-Thiadiazoles Showing the ESIPT Effect Strongly Enhanced by Aggregation

**DOI:** 10.3390/molecules30030531

**Published:** 2025-01-24

**Authors:** Edyta Chruściel, Lidia Ślusarczyk, Bożena Gładyszewska, Dariusz Karcz, Rafał Luchowski, Aleksandra Nucia, Tomasz Ociepa, Michał Nowak, Krzysztof Kowalczyk, Adam Włodarczyk, Mariusz Gagoś, Sylwia Okoń, Arkadiusz Matwijczuk

**Affiliations:** 1 Department of Biophysics, Faculty of Environmental Biology, University of Life Sciences in Lublin, Akademicka 13, 20-950 Lublin, Poland; edyta.chrusciel@gmail.com (E.C.); lidia.slusarczyk@up.lublin.pl (L.Ś.); bozena.gladyszewska@up.lublin.pl (B.G.); 2 Department of Chemical Technology and Environmental Analytics (C1), Faculty of Chemical Engineering and Technology, Cracow University of Technology, Warszawska 24, 31-155 Kraków, Poland; dariusz.karcz@pk.edu.pl; 3 Department of Biophysics, Institute of Physics, Maria Curie-Sklodowska University, 20-031 Lublin, Poland; rafal.luchowski@mail.umcs.pl; 4 Institute of Plant Genetics, Breeding and Biotechnology, University of Life Sciences in Lublin, 20-950 Lublin, Poland; aleksandra.nucia@up.lublin.pl (A.N.); tomasz.ociepa@up.lublin.pl (T.O.); michal.nowak@up.lublin.pl (M.N.); krzysztof.kowalczyk@up.lublin.pl (K.K.); 5 Department of Organic Chemistry and Crystallochemistry, Institute of Chemical Sciences, Faculty of Chemistry, Maria Curie Skłodowska University in Lublin, Gliniana 33, 20-614 Lublin, Poland; adam.wlodarczyk@mail.umcs.pl; 6 Department of Cell Biology, Maria Curie-Sklodowska University, Akademicka 19, 20-033 Lublin, Poland; mariusz.gagos@mail.umcs.pl

**Keywords:** biological properties, biotrophic fungi, imino-1,3,4-thiadiazole derivatives, dual fluorescence effects, ESIPT + AIE

## Abstract

There is currently a growing interest in imino derivatives of compounds such as thiadiazoles and other groups of compounds whose extended π-electron systems enhance their photophysical properties. These compounds also show low toxicity and strong antifungal activity, making them effective against fungal pathogens in crops. For the above reasons, in the first part of the paper, the structure of the selected analogs was considered, and detailed spectroscopic analyses were conducted focusing on the excited state intramolecular proton transfer (ESIPT) process taking place in the same. Measurements were taken in terms of absorption spectroscopy and electron fluorescence, synchronous spectra, and fluorescence lifetimes, as well as calculations of fluorescence quantum efficiency in selected solvents and concentrations. In the spectral observations, the ESIPT process was manifested in several solvents as very distinct dual fluorescence. Moreover, in selected molecules, this phenomenon was strongly related to molecular aggregation, which was associated with not very efficient but nonetheless visible fluorescence of the AIE (Aggregation-Induced Emission) type. In the second part of the paper, a detailed preliminary study is presented exploring the microbiological properties of selected imino-1,3,4-thiadiazole derivatives in the context of their potential applicability as inhibitors affecting the development and growth of some of the most important fungal pathogens attacking cereal crops and posing an increasing threat to modern agriculture. Overall, the research presented in this article provides a detailed, experimental analysis of the spectroscopic properties of selected imino-thiadiazoles and points to their potential use as novel and effective solutions capable of limiting the growth and development of fungal pathogens in cereals.

## 1. Introduction

Producing enough food for the growing human population and keeping up with the increasingly strict regulations regarding consumer and environmental safety are some of the greatest challenges faced by modern agriculture [1,2,3]. More than 50% of the world’s daily caloric intake is supplied directly by cereal grains. The most widely grown cereals include wheat, maize, barley, rye and oat [4]. One of the most important factors endangering the quality and quantity of cereals is the threat of disease caused by various types of pathogens, among which fungal pathogens are particularly dangerous [5,6,7,8]. Protection of cereals against fungal pathogens is a complex process combining biological, agrotechnical and chemical methods. According to the postulated principles of integrated disease management, the use of chemical methods should be the last line of defense against pathogens [9]. However, in many cases, chemical control tillage proves to be the most effective tool in plant protection [10,11]. The effectiveness of fungicides traditionally used to combat plant diseases has dramatically decreased in recent years. At the same time, improper use of conventional fungicides increased the resistance of fungal strains to their active substances [12]. Therefore, it is very important to look for new (innovative) substances that can replace the current therapeutic strategies in combating plant diseases [1,10,11,13,14].

In recent years, the development of heterocyclic agrichemicals has become the leading trend in related research, driven by the compounds’ flexible structure and low toxicity to mammals. Numerous reports available in the literature suggest that various derivatives of 1,3,4-thiadiazoles, with different substituents on the thiadiazole ring, exhibit a wide range of biological activities, including insecticidal [15,16], fungicidal [17,18], and herbicidal properties [19,20,21].

However, in order to ensure their most effective use and develop optimum therapeutic dosages, it is important to understand the mechanisms of a given compound’s interactions in the plant-pathogen system. Therefore, it is first necessary to determine the exact molecular properties of compounds exhibiting biological activity, including their spectroscopic and photophysical characteristics. In this context, 1,3,4-thiadiazole derivatives show very interesting spectroscopic, biophysical, and crystallographic properties [22]. The spectroscopic effects described in the aforementioned works often explain the extraordinary biological potential of this class of analogs, or else they are important just because they enable us to determine how a given molecule behaves in a specific environment, which is significant for the determination of its maximum effectiveness. The spectroscopic properties displayed by this class of analogs, which have been investigated by both our own team and other researchers, include the formation of complexes with rare earth metal ions [23,24,25], exit state intramolecular proton transfer (the ESIPT) [26,27,28,29,30] or, increasingly often, a process of atypical fluorescence associated with AIE aggregation [31,32]. Analogs of this group can also be used as laser dyes [33], molecular probes [34] or UV stabilizers [35,36,37,38].

The ESIPT phenomenon, examined by Weller et al. in the 1960s and more recently by many teams of researchers all over the world [39,40,41,42] is extremely important because of its growing use in industry, for example, in the development of new classes of solar cells [43,44], OLED diodes [45], molecular switches/memories, as highly efficient molecular probes in combination with proteins and DNA systems in molecular biology [34,46], and as very effective and interesting dyes for biological applications [47]. New modifications to this effect, for example, the process of proton transfer triggered proton transfer (PTTPT) [48,49], are reported in the literature all the time. Molecules demonstrating the ESIPT coupled with AIE show quite interesting behavior because their efficiency of quantum emission tends to increase in the solid state due to the limitation of one of the most effective pathways of inactivating the excited state by limiting the rotational capabilities of molecules [50,51].

One should note the well-known fact that chromophore systems typically quench emission in aggregated systems or in the solid state, in a process referred to as aggregation-caused quenching (ACQ) [52,53,54]. However, Luo et al. observed quite different behavior of molecules in the group of aromatic siloles characterized by weak fluorescence in solution but uniquely enhanced emission in the aggregated state, which is exactly what the AIE (aggregated-induced emission) means [55,56]. The mechanism of AIE fluorescence has been associated with restriction of intramolecular motions, i.e., RIM [57,58]. Rotation of molecules absorbing the energy of the excited state by using a non-radiative deactivation channel reduces fluorescent effects; however, in the aggregated state, RIM is capable of blocking the non-radiative channel and thereby activating the radiative channel of deactivation, which in consequence increases the emission of the compound [58]. Compounds showing the AIE type of fluorescence are increasingly used in various fields of science, as well as in agriculture and horticulture. Understanding molecular mechanisms in different analogs will contribute considerably to finding links between structural relationships and microbiological properties of new compounds.

Additionally, it is noteworthy that iminothiadiazoles constitute a unique class of chemical compounds wherein the imine group, in combination with the thiadiazole ring, facilitates the study of electronic shifts and other spectroscopic properties resulting from the particular conjugation of the π system. Their chemical structure is also key to the compounds’ antifungal properties, as the imine group often acts as the pharmacophore, allowing specific interactions with biological molecular targets. These unique properties render iminothiadiazoles particularly interesting in the context of both spectroscopic and biological explorations. Moreover, as follows from the literature, iminothiadiazoles show particular potential in combatting fungal pathogens attacking cereals, such as, e.g., *Fusarium* spp., owing to their ability to disrupt key metabolic processes in such microorganisms, which marks them as promising candidates for effective new plant protection agents.

Therefore, given the growing scientific interest in imino derivatives from various compound groups, we decided to investigate selected iminoderivatives of 1,3,4-thiadiazoles in terms of their spectroscopic properties in solvents of varying polarity, as well as biological potential. One of the main goals of the study was to present a preliminary characterization of the phenomenon of excited state intramolecular proton transfer (ESIPT), which, in the studied analogs, enhances the processes associated with molecular aggregation. The second, equally important objective was to determine whether the selected compounds from the group of 1,3,4-thiadiazoles show fungistatic potential against biotrophic fungal pathogens responsible for some of the most dangerous diseases affecting primary cereal crops.

To the best of our knowledge, the following report is the first to discuss said biological aspects in conjunction with the spectral properties of thiadiazole iminoderivatives and to suggest their innovative use in agriculture as potential new pesticides.

## 2. Results and Discussion

### 2.1. Study of Spectroscopic Effects

**Scheme 1:**
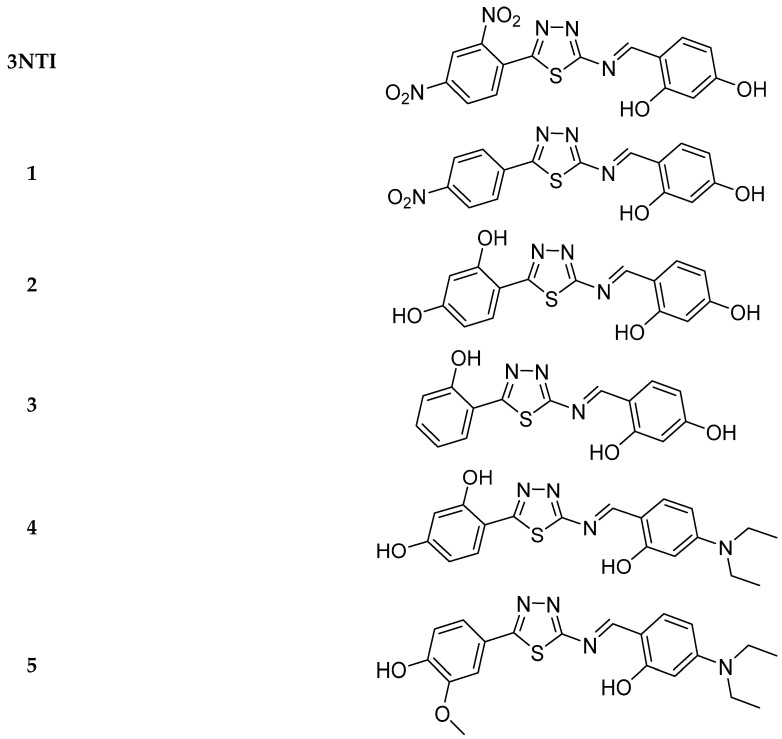
Chemical structures of the compounds selected for the study: (E)-4-(((5-(2,4-dinitrophenyl)-1,3,4-thiadiazol-2-yl)imino)methyl)benzene-1,3-diol (**3NTI**) and five microbiologically tested analogs of imino-1,3,4-thiadiazoles: (E)-4-(((5-(4-nitrophenyl)-1,3,4-thiadiazol-2-yl)imino)methyl)benzene-1,3-diol (1), (E)-4-(5-((2,4-dihydroxybenzylidene)amino)-1,3,4-thiadiazol-2-yl)benzene-1,3-diol (2), (E)-4-(((5-(2-hydroxyphenyl)-1,3,4-thiadiazol-2-yl)imino)methyl)benzene-1,3-diol (3), (E)-4-(5-((4-(diethylamino)-2-hydroxybenzylidene)amino)-1,3,4-thiadiazol-2-yl)benzene-1,3-diol (4), (E)-4-(5-((4-(diethylamino)-2-hydroxybenzylidene)amino)-1,3,4-thiadiazol-2-yl)-2-methoxyphenol (5).

**Scheme 2:**
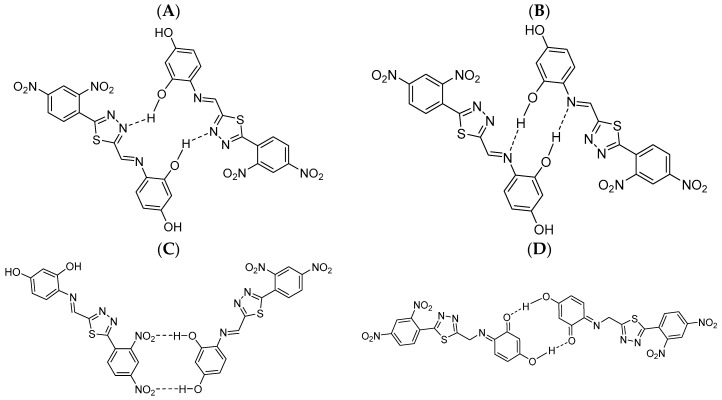
Examples of possible formation of aggregated structures by the **3NTI** analog. Panel (**A**): card pack I, Panel (**B**): card pack II, Panel (**C**): card pack III, Panel (**D**): head to tail.

The molecules of imino-1,3,4-thiadiazole derivatives selected for the study (Scheme 1) contain in their structure a 1,3,4-thiadiazole ring, characteristic of this group of compounds, which connects via the imino group with a resorcylic system. On the opposite side, there are various substituent groups (Scheme 1 and Scheme 2). Some of the compounds selected for this study are also comprised of two distinct sources in their structure, enabling the transfer of a proton in the excited state through hydrogen bonds. One is located on the side directly connected to the resorcylic system, and the other is on the side of the substituent (compounds **2**, **3**, and **4**). The substituents used, as well as the overall structure of the molecules, largely determine the polarity of a given analog.

The analog **3NTI** was chosen for detailed spectroscopic tests. Apart from the arguments given above (in Section 1), the choice of this analog was also supported by the structure of its molecule, in which there is only one possibility of obtaining excited tautomeric forms. Moreover, the compound revealed considerable potential in terms of inhibiting the growth and development of fungal pathogens, as discussed in detail in the second part of the paper.

#### 2.1.1. Stationary Spectroscopy of Electron Absorption and Fluorescence

**Figure 1:**
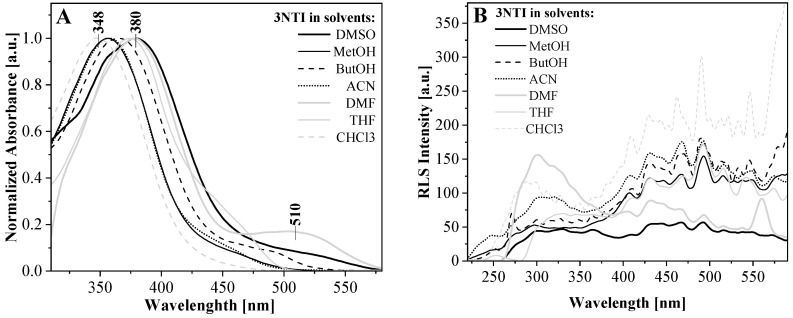
Panel (**A**) shows electron absorption spectra for **3NTI** in the several chosen solvents of different polarities. Panel (**B**) shows examples of waveforms of resonant light scattering RLS for several solvents analogously to Panel (**A**).

Figure 1A shows normalized electron absorption spectra for **3NTI** obtained in several solvents of different polarities. Data on the exact location of electron absorption peaks for all the solvents used in the study are shown in Table S1. As the polarity of a given solvent increases, a fairly large bathochromic shift (Δλ = 32 nm (~2420 cm^−1^) of the band, from 348 nm to 380 nm, can be observed. No such effect is noted for the other 1,3,4-thiadiazoles, but here, it can be explained by quite a long chromophore arrangement of the molecule, which significantly affects the delocalization of the electrons. Thus, the sensitivity of the compound to a change in the polarity of the medium used is evident [59]. Such a strong solvatochromic effect is also linked to an additional polar imine group in the analog’s structure, as well as its high tendency to aggregate. For the electron absorption spectra presented herein, the broad band can be observed from ~330 nm to 440 nm, with the maximum within the range from ~350 to ~380 nm, associated with the main electron transition S_0_→S_1_ in the molecule, which, according to literature, is characteristic for the π→π* electron transition.

At the same time, for most solvents, especially polar ones, we can observe on the longwave side of the main absorption spectral peak a visible and distinct band with a maximum stretching from ~450 to ~520 nm, depending on the solvent. Its clearly weaker intensity, as well as the evident bathochromic shift, in line with the excitonic fission theory, is evidence that various forms of **3NTI** aggregated molecules are present in the given chromophore system (Figure 1A) [60,61,62,63]. This tendency is further confirmed by the significant solvatochromic shift of the main absorption maximum, which is strongly influenced by the molecule’s structure and is conducive to aggregation interactions, for example, through hydrogen bonding [64]. The absorption from dimers or N-aggregates is observed at slightly longer wavelengths than the corresponding imino-1,3,4-thiadiazole, as could be expected from the extension of the delocalized electron system. If the spectral enhancement occurs on the shortwave side, this is the “head to tail” aggregation (Scheme 2A,B), which is just as likely to occur because of the structure of a **3NTI** molecule [63,65]. However, due to the structure of this molecule, there are far more aggregation effects possible, and some other cases are shown in Scheme 2C,D.

In turn, using the relationship:

(1) $$R_{\beta }=\sqrt[{3}]{\frac{\mu^{2}\kappa }{\eta^{2}\beta }}$$

where μ is the dipole moment of transition of the interacting molecules (calculated on the basis of the molecule’s electronic absorption spectra in the given solvent), *η* is the refractive index, *β* is the dipole-dipole interaction energy (in the classical approach, obtained from the difference between the maxima of the emission spectrum and the corresponding absorption spectrum in the given solvent), *κ* = 1 for the card pack molecule arrangement in the aggregate, *κ* = −2 for the head to tail aggregate (parameters adopted from M. Kasha’s exciton splitting theory [64]).

The example distance between the nearest neighbors in the card pack system (obtained from the above equation) is ~3.32Å (Scheme 2A,B). This distance is comparable to those obtained for other similar fluorophores from the group of 1,3,4-thiadiazoles [64].

Having confirmed the strong aggregation effects, the natural subsequent step of the spectral analysis is to measure RLS (Resonance Light Scattering) synchronous spectra. Obtaining RLS spectra for **3NTI** corresponding to the selected spectra from Figure 1A and taking into consideration the research by Pasternak and Colins [65], who linked the occurrence of RLS spectra directly to the presence of aggregated forms of the analyzed compound (such as dimers or larger N-aggregates), we can see in Figure 1B that intensive RLS bands appear practically in all the solvents used. It is worth emphasizing that the oscillatory structure of RLS bands and the solvent-dependent change in intensity in respective spectral regions attest to the possibility of the simultaneous presence of several aggregated forms in a given medium. This is confirmed by the aforementioned structure of the molecule itself and the different possibilities of its aggregation, shown in Scheme 2.

**Figure 2:**
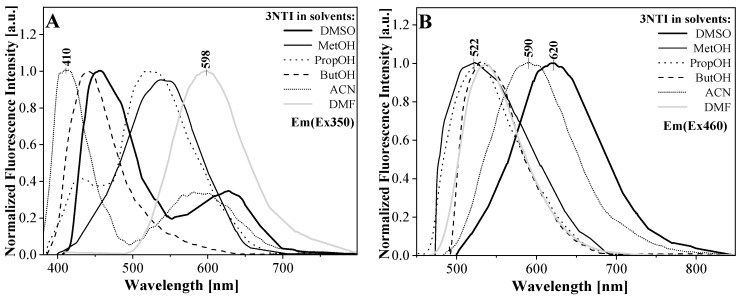
Fluorescence emission spectra corresponding to electron emission spectra from Figure 1. Panel (**A**) shows the excitation in the band’s peak, and Panel (**B**) shows the excitation in the band’s peak combined with aggregation effects.

However, the most interesting effect in the spectral (spectroscopic) context presented in this part of our article is shown in Figure 2 (Panel A), presenting the fluorescence emission spectra normalized at the maxima registered in the chosen solvents. Panel B in the same figure shows the results of measurements of fluorescence emission for the same solvents, but at further excitation, at the maximum corresponding to the aggregated forms (Figure 1A). All the results plotted in this figure, in both panels, were obtained for small concentrations of the compound in a given solvent, approximately ~10^−5^ M, analogously to Figure 1. It can be noticed that depending on changes in the solvent’s polarity, there is a considerable shift in the fluorescence emission band. Emission spectra recorded in solvents, such as DMSO, CAN, or propan-2-ol, after excitation at the maximum of the main absorption band clearly reveal the effect of dual fluorescence in response to a single excitation. This effect depends on the solvent in which it is observed; the first emission band occurs at ~430–450 nm, and the second, for example, in propan-2-ol, peaks at ~520 nm.

In turn, in solvents such as ACN, the first band’s maximum is higher, and the second maximum is shifted much further to ~590 nm. Thus, a strong Stokes shift for longwave emission can be seen, indicating a significant change in the electronic structure of the emitting excitation state [66]. A change in the polarity of the medium leads to the emergence of dual fluorescence. Panel B shows fluorescence emission spectra after excitation at the maximum characteristic for the various **3NTI** aggregated forms. In this case, we can notice a large bathochromic shift of the emission band alongside a change in the polarity of the medium, but there is only a single emission. It is worth noting that the emission illustrated by the spectra in Panel B occurs mostly in the same region as the longwave emission shown in Panel A. This evidences the fact that this emission originates from the same excited molecular form of the given compound.

The structural characteristics of the **3NTI** molecule selected for this study (and other analogs not discussed in this paper), as well as data available from numerous literature reports [56,67,68,69,70,71,72,73,74,75] and our previous studies on similar analogs originating from the group of 1,3,4-thiadiazoles, suggest that the process responsible for the observed fluorescence effects associated with the phenomenon of dual fluorescence is ESIPT (excited state intramolecular proton transfer) [26,76,77,78,79]. The shortwave emission is more likely related to the enol-form emission, whereas the longwave emission seems related to the keto-form emission, occurring only in the excited state [66] (Scheme 2D). Given the specific structure of the molecule, the solvent-dependent fluctuations in the location of the emission band peaks suggest that there could be other molecular forms present, such as *cis*- and *trans*- enol, which may additionally interact with other molecules of the solvent, depending on its character, or with other molecules of a given fluorophore. The evident weakness of the emission, especially in its dual form, indicates that the ESIPT process in the **3NTI** molecule also largely depends on molecular aggregation effects [80] (Scheme 2), which will be the subject of future considerations. This assertion is confirmed by the resonance light scattering (RLS) bands, shown in Figure 1B. They occur where both single and dual fluorescence are observed, implicating that the aggregation process alone does not explain the dual emission in **3NTI**. On the other hand, the distinct RLS bands confirm that aggregation plays a considerable role in the observed effect of dual emission. In fact, what we observe here is strong “cooperation” between ESIPT and aggregation. As follows from the literature and results of our earlier studies, aggregation considerably lowers the energy barrier to the tautomeric transfer between enol* and keto* forms [76,81,82,83,84], thereby facilitating ESIPT, even in media where we do not normally observe it during spectral assays, as is the case with **3NTI**.

#### 2.1.2. Spectroscopic Studies of Concentration Effects

**Figure 3:**
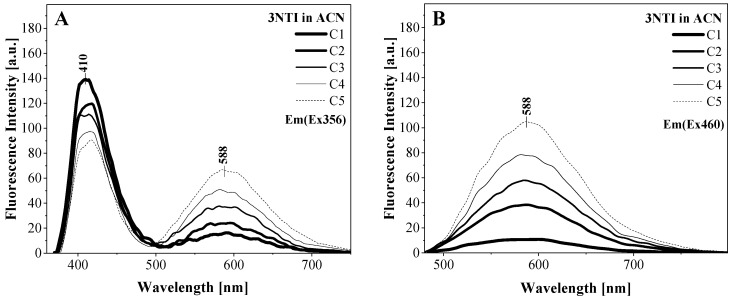
Panel (**A**) shows the fluorescence emission spectra for **3NTI** in ACN in different amounts of the compound (C1-20 μL, C2-26 μL, C3-32 μL, C4-38 μL, and C5-44 μL) at excitation in the maximum of the absorption band. Panel (**B**) shows the analogous spectra of fluorescence emission as in Panel (**A**) at the excitation in the maximum of the aggregation band.

**Figure 4:**
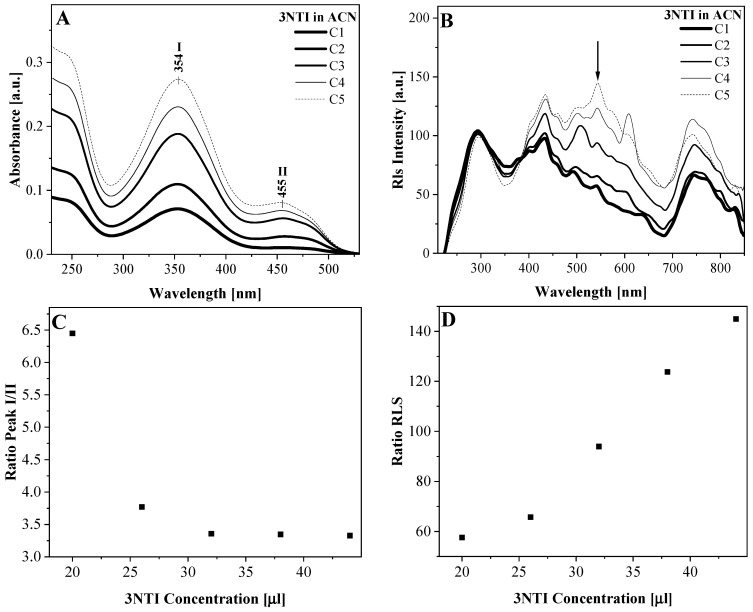
Panel (**A**) presents electron emission spectra for **3NTI** in ACN in different amounts of the compound. Panel (**B**) presents examples of the courses of resonance light scattering RLS spectra for the spectra corresponding to those in Panel (**A**). Panel (**C**) shows absorbance ratios at 354 nm to 455 nm. Finally, Panel (**D**) illustrates an increase in the RLS signal intensity with the maximum at 550 nm depending on the amount of **3NTI**.

The subsequent step in our research entailed a more thorough examination of the impact of aggregation on the effect of dual fluorescence in the **3NTI** molecule. Panels A and B in Figure 3 present spectra of fluorescence emission at two different excitation levels corresponding to the maxima of the absorption spectra, namely at 350 nm and 460 nm (Figure 4A) (denser titration steps for this experiment are presented in Supplementary Materials in Figures S1 and S2). The spectra were captured in ACT, and there was an increase in the concentration of the tested analog in the solution. This led to a very interesting observation. As the concentration of **3NTI** increases, we are able to record dual fluorescence, even at very small increments of the compound’s concentration. While the **3NTI** concentration increases, the first emission band slightly loses in intensity while the second gains considerably. It needs to be mentioned that the concentration of **3NTI** was increased at very small increments so as to avoid any processes (e.g., reabsorption) that might interfere with the course of the experiment in the chosen setup. Panel B shows the results of the measurements in the longwave absorption band maximum. Here, we can observe quite a significant increase in the intensity of fluorescence, which clearly indicates the contribution of processes connected to AIE fluorescence and, therefore, molecular aggregation. Notably, the chosen molecule has many possibilities to form aggregated structures via hydrogen bonds, some of which are shown above in Scheme 2 [85]. Panel D in the above scheme shows possibilities of ketone tautomer aggregation, which in this case is also an acceptable effect. In order to supplement the above results, Figure 4B presents resonance light scattering RLS spectra, which indicate that the intensity of the relevant region increases considerably at ~550 nm. In turn, the intensity of RLS bands with the maximum at ~300 nm remains practically unchanged. Figure 4D presents the ratio of RLS bands’ intensities at 550/300 nm. Clearly, this ratio begins to increase very rapidly with growing concentration. In addition, Figure 4A shows the corresponding spectra of electron absorption registered by our experiment. Here, we observe a large increase in absorbance with the longwave maximum. Figure 4A presents the ratio of absorbance levels at 354 to 455 nm. It is evident that this parameter is practically linear within the given range of concentrations and begins to slightly diverge from linearity only at higher concentrations, which attests to the strong influence of aggregation processes.

In turn, the main band with a maximum at 354 nm is stronger on the longwave side, which, as follows from the exciton fission model and the above discussion, also suggests the type of possible aggregates that are formed at the first moment. Therefore, aggregation facilitates the keto-enol transformation [66] in a given molecule. This effect is reported in the literature and has been investigated for several years as the Aggregation-Induced Emission Enhancement (AIEE) model [80] and has been reported to emerge in an increasing number of molecules [86]. In our future studies focusing on further detailed analysis of spectral effects, we intend to continue exploring this topic. To this end, we plan to synthesize new, targeted test analogs that will allow us to gain more in-depth knowledge of the processes discussed above.

#### 2.1.3. Lifetimes of Fluorescence and Quantum Efficiency

**Figure 5:**
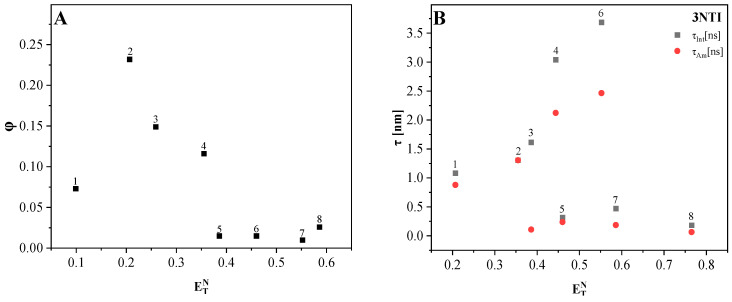
Panel (**A**) shows the dependence of fluorescence quantum efficiency on the solvent’s polarity, where 1—Toluene, 2—THF, 3—Chloroform, 4—Acetone, 5—DMF, 6—ACN, 7—Isopropanol, 8—Butanol. Panel (**B**) shows the dependence of fluorescence lifetime on the solvent’s polarity, where: 1—THF, 2—Acetone, 3—DMF, 4—DMSO, 5—ACN, 6—Isopropanol, 7—Butanol, 8—MetOH.

The next step in our analysis of spectral data demonstrating the impact of ESIPT-related effects, and especially strong aggregation effects associated with AIE fluorescence, entailed the presentation of spectroscopic results for fluorescence quantum efficiency Φ and fluorescence lifetimes measured with the time-correlated single-proton counting method, in several chosen solvents. Figure 5A shows the results of quantum efficiency for **3NTI** and several solvents, depending on the known polarity function $E_{T}^{N}$. As demonstrated for the solvents where the effect of dual fluorescence is clearly seen in fluorescence emission spectra, the quantum efficiency is demonstrably lower than in solvents for which we mostly observe the fluorescence of the enol form and the prevalence of single fluorescence. However, even in cases where single fluorescence emission occurs, we still observe fairly low quantum efficiency of the fluorescence for this compound, which made the analysis presented in this article more demanding. These results again attest to the effect of molecular aggregation on the observed spectral properties of this compound. As known from the literature, the quantum efficiency of fluorescence should typically be higher for polar than for non-polar solvents; however, for **3NTI**, because of its strong preference for aggregation effects, this picture is less clear [66].

**Figure 6:**
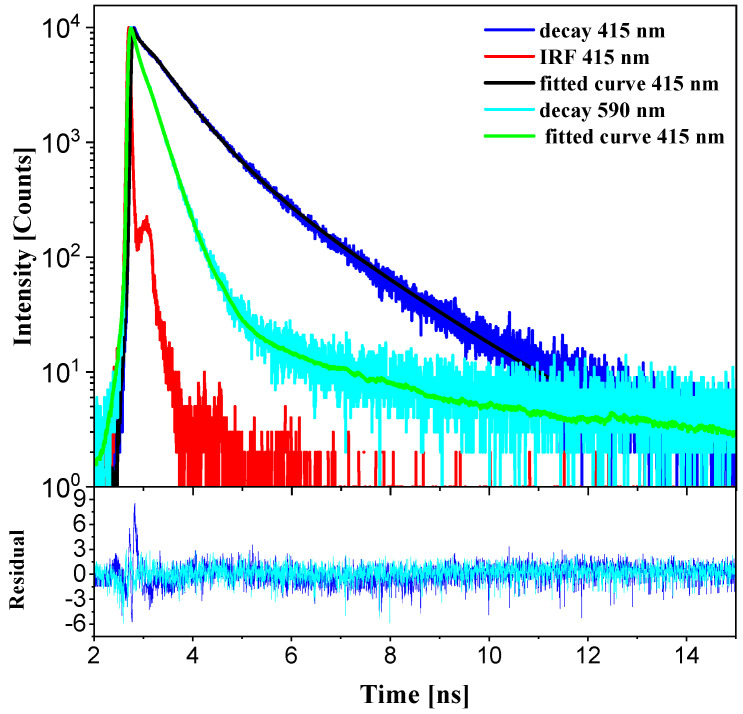
Average fluorescence lifetimes of **3NTI**, dissolved in acetonitrile, measured with a 405 nm diode excitation and observation at 415 nm and 590 nm.

Subsequently, in the following step of our investigation on the ESIPT effect, and particularly its influence on aggregation effects, the corresponding fluorescence lifetimes were measured. The focus was on measuring fluorescence lifetimes in the respective solvents, and their analysis enabled us to detect two main components in the decay curve, characteristic of the possible tautomeric forms related to ESIPT. The detailed results are collated in Table 1. The characteristic decay curves are shown in Figure 6. Figure 5B illustrates the dependence of the obtained lifetimes on the polarity function E_T_(30). This figure shows both lifetimes measured in amplitude and intensity. Clearly, when analyzing these results, we notice that the effect related to aggregation appears to be very strong for practically all the solvents, as discussed above [87]. The presence of two characteristic components of fluorescence lifetime is observed in most of the measurements (Table 1), which is linked to ESIPT [88], where the tautomeric balance between the two forms should be observable even if no visible dual fluorescence effect is detected in the emission spectrum [80].

In the case of the **3NTI** molecule, however, aggregation effects considerably blur the unambiguous division of the components obtained for the specific tautomers. Usually, when we observe a single fluorescence band, mainly the long-life component is recorded (Table 1). If the effect of dual fluorescence appears in a fluorescence emission spectrum, we begin to notice bi-exponential distribution in the lifetime analysis [80]. According to the literature, the shorter lifetime component is related to a ketone tautomer*, and the longer-lifetime component should be assigned to the excited enol tautomer* [89]. Clearly, in virtually every solvent, we can observe the bi-exponential distribution of the fluorescence lifetimes, and the percentage of short- and long-life components depends on the type of solvent. For ACT or isopropanol, this distribution is more classical, although the properties associated with aggregation decisively lengthen a given component and affect differences in the distribution between given tautomers [90].

For comparison with the results obtained for **3NTI** as well as to further highlight the relevance of AIE-enhanced ESIPT effects, Figures S3–S16 and Tables S2 and S3 in the Supplementary Materials present analogous (as well as expanded and comparative) results for analog 2 and 3 (as described in this paper) and **3NTI**. Figures S3–S6 show the electronic absorption spectra, fluorescence emission spectra (for two excitations), and RLS spectra registered for analog 3. The results are fairly analogous to those presented for **3NTI**, particularly in terms of the band enhancement on the longwave side, which evidences the effects of aggregation [60,61,62,63]. In turn, the fluorescence emission spectra clearly reflect the effect of dual fluorescence, which is characteristic of the ESIPT phenomenon. Furthermore, the RLS spectra presented in Figure S6 underscore the influence of aggregation effects, which are clearly present in this iminothiadiazole analog.

Next, by way of further emphasizing the AIE-related aggregation effect, Figures S7–S9 presents the results obtained for analog 3 in an experiment conducted in a THF/water solvent mixture commonly used in numerous reported studies to highlight the effects of AIE fluorescence. As we can see in Figure S7, the absorption spectra show only a slight solvatochromic shift of the bands but Figures S8 and S9 show very radical changes. The emission spectra presented in Figure S8 include a quickly increasing band with the maximum at approx. 450 nm, while the RLS spectra (Figure S9) reveal a significant increase in the intensity thereof.

The subsequent Figures S10–S13 (analog 3), as well as earlier Figures S1 and S2 (**3NTI**), present absorption, fluorescence emission, and RLS spectra (for analog 3), as well and fluorescence emission spectra (for **3NTI**), respectively, registered at concentrations corresponding to those used in the biological studies (presented below). It is interesting and noteworthy, particularly in the context of the presented fluorescence emission spectra, that the effect of dual fluorescence was present at these concentrations. This is particularly notable in the case of **3NTI**, which showed the best microbiological potential (presented below). This effect may be related to the specific structure of the molecule, which in turn might be related not only to the photophysical but also to the biological effects observed. However, no definitive conclusions can be drawn here as further extensive studies would be required to analyze and verify those observations.

Figures S17 and S18, as well as Tables S2 and S3, present the results of fluorescence lifetime measurements obtained in the experiments conducted in solvents as well as the TH/water mixture for analog 3 from the analyzed group of imines. The results clearly indicate two distinct lifetime components, shortwave and longwave, corresponding to the specific tautomeric forms—*keto* and *enol*. This corroborates the results obtained for this analog, as well as for **3NTI**, with the use of stationary methods.

### 2.2. Antifungal Activity

In the subsequent stage of the study, we decided to measure the in vitro antimicrobial activity of each of the obtained derivatives against primary biotrophic pathogens attacking crops at the concentration of 20–5 µg/mL. Detailed results are presented in Table 2.

Powdery mildew caused by *Blumeria* fungi and rust caused by pathogens belonging to the *Pucciniales* order are among the most harmful fungal diseases attacking cereals. These pathogens occur in many regions of the world, causing significant losses in terms of quality and the quantity of crops [91,92,93,94]. The management of fungal diseases currently relies on two primary control practices: the use of resistant cultivars and the application of fungicides. For some crops, commercial cultivars and breeding lines with resistance to powdery mildew and rusts are available [92,95,96,97]; however, the rapid development of new pathogenic strains hinders the effectiveness of resistance breeding [98,99,100].

Therefore, in practice, chemical control, including the use of various chemical fungicides, continues to be the fastest and most effective method of managing fungal diseases [101]. Some of the most common agents used against powdery mildew and rusts in wheat and barley include morpholines (e.g., fenpropidin), demethylase inhibitors (DMIs, e.g., tebuconazole and cyproconazole) and quinone outside inhibitor (QoI, or strobilurin) fungicides [102,103,104]. However, an increasing incidence of adapting, fungicide-resistant pathogens has been observed [105,106]. A good alternative to fungicidal compounds is the use of various types of compounds, which increase plant resistance. Plant activators are compounds that protect cereals against diseases by activating their immune systems; this induces the expression of genes responsible for systemic resistance in the plant [107,108,109]. There are some literature reports claiming that thiadiazoles can be used as compounds with fungicidal activity toward fungal plant pathogens. Giray et al. [110] investigated the effect of thiadiazole derivatives on phytopathogenic fungi: *F. moniliforme*, *F. heterosporum*, *F. culmorum* and *B. cirenea*, concluding that the analyzed compounds effectively inhibited the growth of *F. monilifolie* and *B. cirenea*. Chen et al. [10] analyzed the activity of compounds containing thiadiazole against several different plant pathogens; the authors also demonstrated the possibility of effectively using thiadiazole derivatives in agronomy as agents that inhibit the growth of pathogens. Dai et al. [13,14] provided evidence for the possibility of using thiadiazole compounds as insecticidal, acaricidal and antitumor agents. Given the above, we also aimed to determine whether the obtained 1,3,4-thiadiazole derivatives could indeed be used as factors influencing the growth and development of biotrophic *Blumeria* and *Puccinia* pathogens. In our research, the in vitro tests enabled us to determine the antifungal potential of compounds from the imino-1,3,4-thiadiazole group against the six most important fungal pathogens of the main cereal species (Table 3. Among said six compounds, five showed no effect on the growth and development of the pathogens, regardless of the compound’s concentration in the medium. However, compound **3NTI** was observed to have varied, concentration-dependent inhibitory impact on the growth and development of the pathogens. This compound completely inhibited the growth and development of all the analyzed pathogens at concentrations of 20 and 10 µg/mL. The application of concentrations between 9 and 8 µg/mL made it possible to control the presence of pathogens of the genera *Blumeria* and *Puccina* on all the analyzed cereal species with 90% efficiency. At the lowest tested concentration, i.e., 5 µg/mL, the growth of all the biotrophic pathogens was limited by over 50% relative to the control tested without the application of the compound.

Having applied the analyzed compounds by spraying them on the plant, inhibition of the growth of *Blumeria* and *Puccinia* pathogens was only observed in the presence of **3NTI**, which inhibited the growth of both pathogens with 94–98% effectiveness (Table 2).

Both the addition of the analyzed compounds to the agar medium and the spraying of the plant leaves resulted in similar effects. This may indicate that the compound **3NTI** acts as an inducer of plant resistance. However, this hypothesis needs to be verified by conducting further studies, including an analysis of gene expression and plant protein profiles after the application of the analyzed compounds, which will be explored in our future research.

To briefly summarize the above observations, it should be reiterated that understanding the mechanisms underlying the interactions of active compounds in plant-pathogen systems is a crucial factor facilitating the development of new agents for agriculture. The first step in such studies should entail precise molecular characterization of relevant compounds. Given the above, this article presents a study on the spectroscopic and photophysical properties of the analyzed compounds, which, due to their unique structure, constitute a particularly interesting object of research. To this end, preliminary photophysical investigations were conducted (presented above), focusing in particular on the **3NTI** derivative showing considerable fungicidal potential, as demonstrated in our preliminary biological studies. For the sake of comparison, parallel experiments were also done for other compounds (**3** and to some extent **2**), which did not, however, demonstrate biological activity in inhibiting the growth and development of biotrophic fungal pathogens.

In our future research, we intend to attempt more in-depth identification of the relevant properties of the analyzed compounds and their potential relationship with the induction of plant resistance. However, further advancement in this area of research will require the use of advanced molecular biology techniques and considerable financial outlays, which may prove challenging for obvious reasons.

## 3. Materials and Methods

The subject of the study consisted of imino derivatives of 1,3,4-thiadiazoles, shown in Scheme 1.

### 3.1. Synthesis of Thiadiazole Derived Imine

**Scheme 3:**
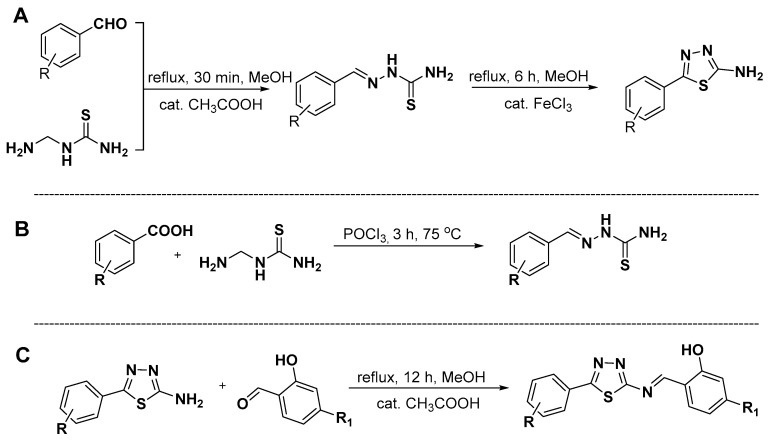
Synthetic pathway of the synthesis of thiadiazole-derived imines **3NTI** and **1**–**5** (Route (**A**) then (**C**) was employed for compounds **3NTI** and **1**; route (**B**) then (**C**) was employed for compounds **2**–**5**.

Two classical synthetic routes were employed to isolate 1,3,4-thiadiazole intermediates. The first involved the formation of thiosemicarbazone and its subsequent oxidative cyclization in the presence of excess FeCl_3_ (Scheme 3A). In particular, the equimolar amount of thiosemicarbazide (Merck, Darmstadt, Germany) and appropriate benzaldehyde were refluxed for 30 min in methanol (Avantor, Gliwice, Poland) with a catalytic amount of glacial acetic acid (Merck Darmstadt, Germany) (2,4-dinitrobenzaldehyde (Merck, Darmstadt, Germany), 4-nitrobenzaldehyde (Merck, Darmstadt, Germany) were used for the synthesis of **3NTI** and **1** respectively). The mixture was then cooled down, and the resulting thiosemicarbazone was filtered off, washed with cold methanol, and dried [111,112]. The thiosemicarbazone was then refluxed in ethanol with a 3-fold excess of FeCl_3_·6H_2_O (Warchem, Zakręt, Poland) for 6 h, cooled down and neutralized with the use of 30% sodium thiosulfate (Warchem, Zakręt, Poland) (aq). The precipitate formed was filtered off, dried, and recrystallized from 50% ethanol (Honeywell, Charlotte, NC, USA) (aq) [111,113]. The second synthetic route was based on our previously published procedure [114] involving the POCl_3_ (Merck, Darmstadt, Germany) -catalyzed reaction of the appropriate substituted benzoic acid and thiosemicarbazide, yielding the hydroxyphenyl-substituted 1,3,4-thiadiazoles (Scheme 3B). Compounds **2**–**5** were obtained using this method. The thiosemicarbazone and 2-aminothiadiazole intermediates obtained in this work are known, and hence, their detailed structural elucidation was omitted. All the 2-amino-1,3,4-thiadiazoles obtained were subsequently reacted with the appropriate salicylaldehydes, yielding the corresponding imines (Scheme 3C). This procedure has been previously reported, also by our group [115] and involves refluxing the equimolar amount of the thiadiazole intermediate and the appropriate substituted salicylaldehyde in methanol in the presence of a catalytic amount of glacial acetic acid (2,4-dihydroxybenzaldehyde was used for the synthesis of **3NTI**, **1**–**3** while 2-hydroxy-4-diethylaminobenzaldehyde was used in the synthesis of **4** and **5**). The precipitate formed upon cooling the reaction mixture to ambient temperature was filtered off and recrystallized from absolute ethanol. A single crystal of compound **5** suitable for the XRD analysis [116,117] was isolated by slow evaporation of the ethanolic solution. The yields and the spectroscopic data for all imines obtained are provided in the Supplementary Materials Figures S19–S37.

### 3.2. Antifungal Activities

Antifungal activities were tested in vitro against the main biotrophic fungal pathogens attacking cereals (Table 3).

The target compounds were dissolved in DMSO to obtain a stock solution of 100ug/mL. Two strategies were used to evaluate the effect of compounds on the pathogens’ growth. In the first, compounds were evaluated using the following dilutions: 20, 10, 9, 8, 7, 6 and 5 ug/mL. An appropriate amount of the compound was added to 10 mL of agar medium (6 g/L) in order to obtain the desired concentrations. Leaves of the Fuchs (*A. sativa*), Błyskawica (*T. aestivum*), Klaus (*x Triticosecale*), and Mecenas (*H. vulgare*) cultivars susceptible to the analyzed pathogens were placed on Petri dishes with the agar medium and the addition of the analyzed compounds. Control leaves were placed on Petri plates with agar supplemented with DMSO. Petri dishes with leaf fragments were inoculated with pathogen spores according to the host-pathogen test methodology described by Hsam et al. [118]. Each test was performed in five independent replications.

In the second strategy, leaves of the susceptible cultivars were placed on plates filled with agar medium without any additives and then sprayed with a solution containing the analyzed compounds at a concentration of 10 µg/mL. In the first variant, the spraying was performed before the spore inoculation, and in the second, after pathogen inoculation. After 10 days, the infection of the leaves was assessed on a five-point scale presented in Table 4.

The results of the percentage disease control were calculated as compared with the control plants, wherein 100 was rated as complete disease control and 0 as no disease control [10]. The percentage of disease control was presented as a mean value from five independent replications of each solution of a given compound.

### 3.3. Preparation of Compounds for Photophysical Measurements

Stock solutions of **3NTI** and the other tested analogs were prepared by dissolving 1 mg of the compound in selected solvents (all listed in Section 2 below). All the solvents used in this study were of analytical grade. An appropriate volume of the solution was added to 3 mL of solvent in order to obtain the required absorbance intensity. The molar concentration of **3NTI** and the other compounds dissolved in various solutions was C~1.5·10^−3^ M.

#### 3.3.1. NMR

The NMR study focused on the characterization of six compounds from the group of 1,3,4-thiadiazoles (SM) containing an imine bond in position 2, and derivatives of phenol, resorcinol, and nitroarenes substituted with aromatic rings in position 5.

NMR spectra were measured on a Bruker Avance (Karlsruhe, Germany), 500 MHz instrument in DMSO-d6 as the solvent. During preparation, the samples were also dissolved in CDCl_3_ and MeOH-d4, but they dissolved very poorly; hence, DMSO-d6 was chosen as the main solvent, which allowed us to obtain homogeneous solutions with good quality data. Signals were assigned based on standard chemical shifts, as described previously in similar publications [114].

#### 3.3.2. Electronic Absorption and Fluorescence Spectra

Electronic absorption spectra of the selected *imino*-1,3,4-thiadiazole derivatives were registered on a double beam UV–Vis Cary 300 Bio spectrophotometer (Varian, Mulgrave Victoria, Australia), equipped with a 6 × 6 Peltier thermostatted multicell holder. Temperature was controlled with a Carry Series II thermocouple sensor (Varian, Mulgrave Victoria, Australia) placed directly in a sample. The measurements of absorption spectra were taken at room temperature, within the spectral range from 250 to 600 nm.

To record emission spectra, excitations and synchronous spectra, a Cary Eclipse spectrofluorometer (Varian, Mulgrave Victoria, Australia) was employed. All measurements were carried out at room temperature. Fluorescence spectra were recorded at a resolution of 0.5 nm, with corrections of the spectral characteristics of the tube and photomultiplier. Ex 5 and Em 5 spectral slits were used with standard spectrofluorometer voltage. For clarity of presentation, the spectra were correlated using the Savitzky–Golay filter to smooth the lines.

Measurements of Resonance Light Scattering (synchronous spectra) were carried out according to the previously described protocol, with synchronous scanning of both the excitation and emission monochromators (with no interval between excitation and emission wavelengths) at a spectral resolution of 1.5 nm. Ex 2.5 and Em 2.5 spectral slits were used with standard spectrofluorometer voltage. For clarity of presentation, the spectra were correlated using the Savitzky–Golay filter to smooth the lines.

The software used to process the recorded data was Grams/AI 8.0 (Thermo Electron Corporation; Waltham, MA, USA).

#### 3.3.3. Fluorescence Quantum Yield

The quantum yields of fluorescence of **3NTI** solutions were determined using 7-diethylamino-4-methylcoumarin (coumarin1) as the fluorescence standard. The measurements were carried out in ethanol Φ*_F_* = 0.73 [91]. The final fluorescence quantum yield values were calculated from the following Equation (2):

(2) $$\Phi_{F\left(X\right)}=\Phi_{R\left(MeOH\right)}\left(\frac{\lambda_{exR(MeOH)}}{\lambda_{exX}}\right)\left(\frac{I_{X}}{I_{R(MeOH)}}\right)\left(\frac{\eta_{X}^{2}}{\eta_{R(MeOH)}^{2}}\right)$$

where the subscript *X* denotes corresponding **3NTI** in different solvents/ratios, *λ_ex_* is the value of absorbance at the excitation wavelength, *I*—is the plane under the emission curve, and *η* is the refractive index of the solvent.

*R*—coumarin1,

*X*—compound

#### 3.3.4. Fluorescence Lifetimes

Time-resolved fluorescence decays were measured using a fully automated spectrofluorometer (model FluoTime 300, PicoQuant GmbH, Berlin, Germany) following the time-correlated single-photon counting method. Excitation was achieved using a pulsed laser diode (P-C-405B) working at 405 nm (50 ps fwhm time resolution) and 20 MHz repetition rate. Fluorescence decays were recorded using a Micro Channel Plate-PMT detector ombined with temporal acquisition resolution up to 4 ps. Time fluorescence decay modeling was performed with EasyTau v. 1.4. software. For observation, a Czerny-Turner monochromator (Bentham Instruments Ltd., Livingston Village, UK) was used with selected grating blazed at 500 nm and 600 lines/mm. The emission was observed at a magic angle using a Glan-Thompson polarizer (Getynga, Germany), and an additional 405 long-wavelength pass filter (BLP01-405R-25, Semrock, New York, NY, USA). The quality of the fit was assessed by minimizing the reduced chi-squared function (χ^2^_R_) and visual inspection of the weighted residuals. Measurements of fluorescence lifetimes were taken, similarly to other spectroscopic measurements, at room temperature.

## 4. Conclusions

The spectroscopic and lifetime-distribution results obtained for the **3NTI** analog selected based on the results of the biological studies, complemented by the microbiological assays, show that the observed phenomenon of dual fluorescence in the chosen analog from the imino-derivatives of 1,3,4-thiadiazoles is primarily due to the effect of excited state intramolecular proton transfer ESPIT. The experiments were run at different concentrations of **3NTI** in selected organic solvents, and synchronous spectra for the same solvents demonstrated that aggregation has a very strong influence on dual fluorescence, preferring it even in low-concentration environments– the AIE effect. Aggregation means that the solvent-solute interaction is weakened, in addition to lowering the energy barrier to transfer between tautomers. The tested analog’s quantum efficiency is relatively low, mostly because of the effects produced by aggregation-related processes.

In turn, the microbiological tests conducted on selected fungal pathogens affecting cereals show that the compound chosen for the study, **3NTI**, can be used as a very promising inhibitor of the development and growth of biotrophic pathogens from the genera of Blumeria and Puccinia. Moreover, **3NTI** also showed high potential against all the other analyzed pathogens invading various cereal species, which indicates that it might be used against a wide spectrum of diseases. It is, therefore, fully justified to search for further derivatives of 1,3,4-thiadiazole, modified with various substituents capable of enhancing the microbiological activity of this group.

In our future research, we will continue to explore the effects of **3NTI** on plant resistance by studying the expression of genes activated when the plants are exposed to the compound, as well as analyzing the proteome. These studies will allow us to draw more definitive conclusions regarding the way that **3NTI** and other similar compounds influence plants and inhibit the growth and development of fungal pathogens. Detailed studies focusing on the ESIPT process will be supplemented in the future by the inclusion of tests on other analogs from the same group and accompanying quantum efficiency calculations. This combination of biological and biophysical studies will allow us to precisely characterize the activity observed in this type of compound and clarify the relevant mechanism of growth and development inhibition in biotrophic fungal pathogens.

## Figures and Tables

**Table 1:** Examples of fluorescence lifetime for **3TNI** in the selected solvents.

Solvent	τInt [ns]	τAM [ns]	τ1 [ns]	ARel1 [%]	τ2 [ns]	ARel2 [%]	τ3 [ns]	ARel3 [%]
DMSO in 460 nm	3.0422	2.1228	1.0080	29.0	3.8735	71.0	-	-
Methanol in 540 nm	0.1849	0.0657	0.0606	91.8	1.5830	8.2	-	-
Isopropanol in 460 nm	3.6860	2.4650	4.4820	77.3	0.9730	22.3	-	-
Butanol in 440 nm	0.4735	0.1865	0.2814	32.9	1.2291	27.8	0.0990	39.3
ACN in 590 nm	0.3182	0.2389	0.1045	15.2	0.3033	82.7	2.3960	2.1
DMF in 600 nm	1.6155	0.1087	0.0633	56.8	4.8730	30.5	0.7446	12.7
Acetone in 560 nm	1.3059	1.3059	1.3059	100%	-	-	-	-
THF in 535 nm	1.0834	0.8788	0.8299	93.2	4.5570	6.8	-	-

**Table 2:** Percentage disease control by tested compounds.

Cereal	Pathogen	Compound	Compound Concentration							Spraying Before Infection	Spraying After Infection
			20	10	9	8	7	6	5		
Avena sativa (oat)	*Blumeria graminis* f.sp. *avenae*,	**3NTI**	100	100	94	92	74	62	56	98	96
		**1**	0	0	0	0	0	0	0	0	0
		**2**	0	0	0	0	0	0	0	0	0
		**3**	0	0	0	0	0	0	0	0	0
		**4**	0	0	0	0	0	0	0	0	0
		**5**	0	0	0	0	0	0	0	0	0
	*Puccinia coronata* f.sp. *avenae*	**3NTI**	100	100	100	94	76	62	56	98	98
		**1**	0	0	0	0	0	0	0	0	0
		**2**	0	0	0	0	0	0	0	0	0
		**3**	0	0	0	0	0	0	0	0	0
		**4**	0	0	0	0	0	0	0	0	0
		**5**	0	0	0	0	0	0	0	0	0
Triticum aestivum (wheat)	*Blumeria graminis* f.sp. *tritici*,	**3NTI**	100	100	98	94	74	68	62	98	94
		**1**	0	0	0	0	0	0	0	0	0
		**2**	0	0	0	0	0	0	0	0	0
		**3**	0	0	0	0	0	0	0	0	0
		**4**	0	0	0	0	0	0	0	0	0
		**5**	0	0	0	0	0	0	0	0	0
	*Puccinia recondita* f.sp. *tritici*.	**3NTI**	100	100	100	92	76	56	56	98	96
		**1**	0	0	0	0	0	0	0	0	0
		**2**	0	0	0	0	0	0	0	0	0
		**3**	0	0	0	0	0	0	0	0	0
		**4**	0	0	0	0	0	0	0	0	0
		**5**	0	0	0	0	0	0	0	0	0
Hordeum vulgare (barley)	*Puccinia hordei*	**3NTI**	100	100	98	96	78	68	62	98	96
		**1**	0	0	0	0	0	0	0	0	0
		**2**	0	0	0	0	0	0	0	0	0
		**3**	0	0	0	0	0	0	0	0	0
		**4**	0	0	0	0	0	0	0	0	0
		**5**	0	0	0	0	0	0	0	0	0
Triticosecale (triticale)	*B. graminis* f.sp. *triticale*	**3NTI**	100	100	96	90	70	62	56	98	96
		**1**	0	0	0	0	0	0	0	0	0
		**2**	0	0	0	0	0	0	0	0	0
		**3**	0	0	0	0	0	0	0	0	0
		**4**	0	0	0	0	0	0	0	0	0
		**5**	0	0	0	0	0	0	0	0	0

**Table 3:** Pathogens and their hosts used in the study.

Cereal	Pathogen	Disease
oat	*Blumeria graminis* f.sp. *avenae*,	powdery mildew
	*Puccinia coronata* f.sp. *avenae*	crown rust
wheat	*Blumeria graminis* f.sp. *tritici*,	powdery mildew
	*Puccinia recondita* f.sp. *tritici*.	brown rust
barley	*Puccinia hordei*	leaf rust
triticale	*B. graminis* f.sp. *triticale*	powdery mildew

**Table 4:** A five-point scale for assessing oat leaf infection by fungal pathogens.

The Degree of Infection	Description of the Degree	Percentage Disease Control
0	Lack of symptoms	100%
1	Limited development of the pathogen visible as single and small colonies	90%
2	Mycelium visible with a small quantity of spores—less than 20% of the leaf area	80%
3	Extensive mycelium occupying 20–50% leaf area	50%
4	Abundant mycelium occupying more than 50% of the leaf surface	0%

## Data Availability

Data is contained within the article or Supplementary Materials.

## Supplementary Materials

The following supporting information can be downloaded at: https://www.mdpi.com/article/10.3390/molecules30030531/s1, Table S1. Function values $E_{T}^{N}$, electronic absorption maxima, fluorescence emission spectral maxima, Stokes shift values and quantum yields for **3NTI** in selected solvents; Schemes NMR results, Figure S1. Fluorescence emission spectra for **3NTI** at concentrations corresponding to the biological results, at the wavelength corresponding to the main absorption spectrum maximum, Figure S2. Fluorescence emission spectra for **3NTI** at concentrations corresponding to the biological results, at the wavelength corresponding to aggregated structures, Figure S3. Electronic absorption spectra for analogue no. 3 in selected solvents of varying polarity, Figure S4. Fluorescence emission spectra for analogue no. 3, corresponding to the spectra shown in Figure S1, at a greater excitation wavelength, Figure S5. Fluorescence emission spectra for analogue no. 3, corresponding to the spectra shown in Figure S1, at a shorter excitation wavelength, Figure S6. Resonance light scattering spectra for analogue no. 3, corresponding to the spectra Show in Figure S1, Figure S7. Electronic absorption spectra for analogue no. 3, in the solvent mixture THF:H_2_O, Figure S8. Fluorescence emission spectra for analogue no. 3 in the solvent mixture THF:H_2_O, corresponding to those shown in Figure S5, Figure S9. RLS spectra for analogue no. 3 in the solvent mixture THF:H_2_O, corresponding to those shown in Figure S5, Figure S10. Electronic absorption spectra for analogue no. 3 at concentrations corresponding to the biological results, Figure S11. Fluorescence emission spectra for analogue no. 3 at concentrations corresponding to the biological results, Figure S12. Fluorescence emission spectra for analogue no. 3 at concentrations corresponding to the biological results, at wavelengths corresponding to aggregated structures, Figure S13. RLS spectra for analogue no. 3 at concentrations corresponding to the biological results, Figure S14. Electronic absorption spectra for analogue no. 2 in the several chosen solvents of different polarity, Figure S15. Fluorescence emission spectra for analogue no. 2 corresponding to electron emission spectra from Figure S14, Figure S16. RLS spectra for analogue no. 2, Table S2. Examples of fluorescence lifetime measurements for analogue 3 in the selected solvents, Table S3. Examples of fluorescence lifetime for analogue 3 in the solvent mixture THF:H_2_O, Figure S17. Example fluorescence lifetime curves in selected solvents, for analogue no. 3, Figure S18. Example fluorescence lifetime curves for analogue no. 3, in solvent mixture THF:H_2_O, Figure S19. ^1^H NMR Spectra of **3NTI**, Figure S20. 1H NMR Spectra of 1, Figure S21. ^1^H NMR Spectra of 2, Figure S22. ^1^H NMR Spectra of 3, Figure S23. ^1^H NMR Spectra of 4, Figure S24. ^1^H NMR Spectra of 5, Figure S25. Molecular structures of (E)-4-(5-((4-(diethylamino)-2-hydroxybenzylidene)amino)-1,3,4-thiadiazol-2-yl)-2-methoxyphenol observed in the solid. The thermal ellipsoids are drawn at a 50% probability level, Figure S26. ^1^H NMR Spectra of **3NTI**, Figure S27. ^13^C NMR Spectra of **3NTI**, Figure S28. ^1^H NMR Spectra of 1, Figure S29. ^13^C NMR Spectra of 1, Figure S30. ^1^H NMR Spectra of 2, Figure S31. ^13^C NMR Spectra of 2, Figure S32. ^1^H NMR Spectra of 3, Figure S33. ^13^C NMR Spectra of 3, Figure S34. ^1^H NMR Spectra of 4, Figure S35. ^13^C NMR Spectra of 4, Figure S36. ^1^H NMR Spectra of 5, Figure S37. ^13^C NMR Spectra of 5.
