# Supplementary material for: Assessment of the Microbiological Potential and Spectroscopic Properties of New Imino-1,3,4-Thiadiazoles Showing the ESIPT Effect Strongly Enhanced by Aggregation
Source: Molecules. 2025 Jan 24;30(3):531. doi: 10.3390/molecules30030531 (PMC11820074; doi:10.3390/molecules30030531)
Supplement: Supplementary file 1 [file molecules-30-00531-s001.zip › molecules-3390093-supplementary.pdf]

Article

Assessment of the Microbiological Potential and Spectroscopic Properties of New Imino-1,3,4-Thiadiazoles Showing the ESIPT Effect Strongly Enhanced by Aggregation

Edyta Chruściel [1], Lidia Ślusarczyk [1], Bożena Gładyszewska [1], Dariusz Karcz [2], Rafał Luchowski [3], Aleksandra Nucia [4], Tomasz Ociepa [4], Michał Nowak [4], Krzysztof Kowalczyk [4], Adam Włodarczyk [5], Mariusz Gagoś [6], Sylwia Okoń [4,*] and Arkadiusz Matwijczuk [1,*]

1 Department of Biophysics, Faculty of Environmental Biology, University of Life Sciences in Lublin, Akademicka 13, 20-950 Lublin, Poland; edyta.chrusciel@gmail.com (E.C.); lidia.slusarczyk@up.lublin.pl (L.Ś.); bozena.gladyszewska@up.lublin.pl (B.G.)
2 Department of Chemical Technology and Environmental Analytics (C1), Faculty of Chemical Engineering and Technology, Cracow University of Technology, Warszawska 24, 31-155 Kraków, Poland; dariusz.karcz@pk.edu.pl
3 Department of Biophysics, Institute of Physics, Maria Curie-Sklodowska University, 20-031 Lublin, Poland; rafal.luchowski@mail.umcs.pl
4 Institute of Plant Genetics, Breeding and Biotechnology, University of Life Sciences in Lublin, 20-950 Lublin, Poland; aleksandra.nucia@up.lublin.pl (A.N.); tomasz.ociepa@up.lublin.pl (T.O.); michal.nowak@up.lublin.pl (M.N.); krzysztof.kowalczyk@up.lublin.pl (K.K.)
5 Department of Organic Chemistry and Crystallochemistry, Institute of Chemical Sciences, Faculty of Chemistry, Maria Curie Skłodowska University in Lublin, Gliniana 33, 20-614 Lublin, Poland; adam.wlodarczyk@mail.umcs.pl
6 Department of Cell Biology, Maria Curie-Sklodowska University, Akademicka 19, 20-033 Lublin, Poland; mariusz.gagos@mail.umcs.pl
* Correspondence: sylwia.okon@up.lublin.pl (S.O.); arkadiusz.matwijczuk@up.lublin.pl (A.M.); Tel.: +48-814-456-920 (S.O.); +48-814-456-937 (A.M.); Fax: +48-814-456-684 (A.M.)

Table S1. Function values E_T^N, electronic absorption maxima, fluorescence emission spectral maxima, Stokes shift values and quantum yields for 3NTI in selected solvents.

Lp.	solvent	E_T^N	λ_{abs} [nm/cm^{-1}]	λ_{abs} [nm/cm^{-1}]	λ_{em} [nm/cm^{-1}]	λ_{em} [nm/cm^{-1}]	φ
1	DMSO	0,444	379 (26385)	525 (19048)	455 (21978)	627 (15949)	-
2	Methanol	0,765	358 (27933)	460 (21739)	-	543 (18416)	-
3	Ethanol	0,654	360 (27778)	467 (21413)	-	530 (18868)	-
4	Isopropanol	0,552	367 (27248)	-	429 (23310)	523 (19120)	0,010

5	Butanol	0,586	365 (27397)	492 (20325)	438 (22831)	-	0,026
6	Acetonitryle	0,460	358 (27933)	455 (21978)	410 (24390)	591 (16920)	0,015
7	DMF	0,386	378 (26455)	510 (19608)	-	598 (16722)	0,015
8	Acetone	0,355	366 (27322)	510 (19608)	-	560 (17857)	0,116
9	Ethyl acetate	0,228	366 (27322)	464 (21552)	-	533 (18762)	-
10	THF	0,207	374 (26738)	460 (21739)	422 (23697)	535 (18692)	0,231
11	Chloroform	0,259	348 (28736)	-	-	554 (18051)	0,149
12	Toluene	0,099	357 (28011)	460 (21739)	-	520 (19231)	0,073

Experimental Details for the synthesized imines:

Synthetic protocol: The equimolar amount of 2-aminothiadiazole and appropriate salicylaldehyde was placed in a round bottom flask and dissolved in methanol. The mixture was then stirred and refluxed overnight. The precipitate formed upon cooling the mixture to ambient temperature was filtered off and recrystallized from absolute ethanol yielding the imine product.

(E)-4-(((5-(2,4-dinitrophenyl)-1,3,4-thiadiazol-2-yl)imino)methyl)benzene-1,3-diol (3NTI)

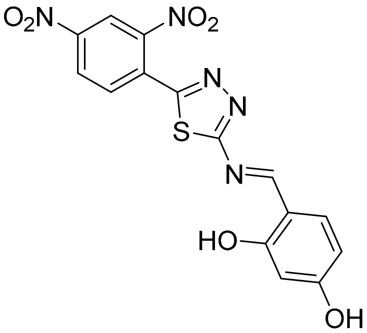

Yield: 65%; ^{1}H NMR (500 MHz, DMSO-d6) δ = 6.31 (br. s., 1 H); 7.35 (br. s., 1 H); 7.64 (br. s., 1 H); 8.05 (d, *J*=6.62 Hz, 1 H); 8.50 (d, *J*=6.31 Hz, 1 H); 8.81 (br. s., 1 H); 9.92 (br. s., 1 H); 10.62 (br. s., 1 H); 10.89 (br. s., 1 H).

^{13}C NMR (126 MHz, DMSO-d6) δ = 102.64 (s), 109.28 (s), 120.25 (s), 127.34 (s), 128.88 (s), 128.92 (s), 132.73 (s), 132.76 (s), 147.70 (s), 147.89 (s), 149.14 (s), 165.71 (s), 171.45 (s), 191.42 (s), 208.51(s).

HRMS Theoretical mass [M+H]+ 388.03518; Experimental mass [M+H]+ 388.03533

(E)-4-(((5-(4-nitrophenyl)-1,3,4-thiadiazol-2-yl)imino)methyl)benzene-1,3-diol (1)

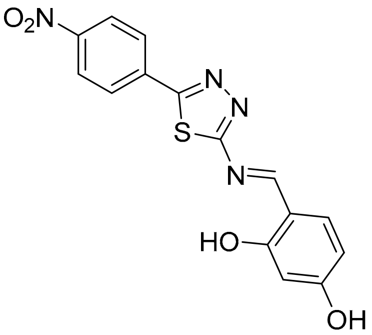

Yield: 72%; ^{1}H NMR (500 MHz, DMSO-d6) δ = 6.30 - 6.34 (m, 1 H) [minor isomer], 6.40 (s) [mixed isomers], 6.45 - 6.52 (m, 1 H) [minor isomer], 7.51 - 7.56 (m, 1 H) [minor isomer], 7.74 (s, 1 H) [mixed isomers], 7.77 (d, *J*=8.83 Hz, 2 H) [mixed isomers], 7.99 - 8.05 (m, 1 H) [minor isomer], 8.24 (d, *J*=8.51 Hz, 1 H) [major isomer], 8.28 - 8.33 (m, 1 H) [minor isomer], 8.40 (d, *J*=8.51 Hz, 3 H) [major isomer], 9.12 (s, 1 H) [major isomer], 9.90 - 9.95 (m, 1 H) [minor isomer], 10.59 - 10.66 (m, 1 H) [minor isomer], 10.79 (s, 1 H) [major isomer], 10.88 - 10.92 (m, 1 H) [minor isomer], 11.53 (s, 1 H) [major isomer] ppm

^{13}C NMR (126 MHz, DMSO-d6) δ =102.83 (s), 110.00 (s), 112.76 (s), 124.91(s), 125.10 (s), 127.57 (s), 128.84 (s), 136.208 (s), 137.34 (s), 137.38 (s), 149.04 (s), 163.71 (s), 165.85(s) ppm.

HRMS Theoretical mass [M+H]+ 343.05010; Experimental mass [M+H]+ 343.05018

(E)-4-(5-((2,4-dihydroxybenzylidene)amino)-1,3,4-thiadiazol-2-yl)benzene-1,3-diol (2)

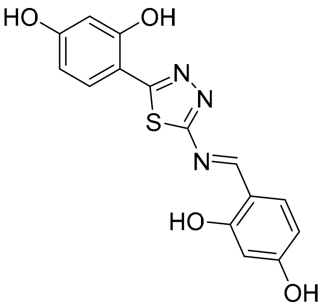

Yield: 43%; ^{1}H NMR (500 MHz, DMSO-d6) δ = 6.30 - 6.41 (m, 1 H) [minor isomer], 6.42 - 6.51 (m, 1 H) [major isomer], 7.12 - 7.20 (m, 1 H), 7.49 - 7.58 (m, 2 H), 7.69 (d, *J*=8.51 Hz, 2 H), 8.00 (d, *J*=8.83 Hz, 2 H), 9.07 (s, 2 H), 9.80 - 9.88 (m, 1 H), 9.93 (s, 1 H), 10.08 (br. s., 1 H), 10.65 (br. s., 1 H), 10.90 (br. s., 1 H), 11.01 - 11.13 (m, 2 H), 11.93 (br. s., 2 H) ppm

^{13}C NMR (126 MHz, DMSO-d6) δ = 102.64 (s) [minor isomer], 102.83 (s) [major isomer], 108.36 (s) [mixed isomers], 108.82 (s) [mixed isomers], 109.05 (s) [mixed isomers], 109.14 (s) [mixed isomers], 109.53 (s)

[mixed isomers], 112.59 (s) [major isomer], 115.68 (s) [minor isomer], 129.09 (s) [major isomer], 129.21 (s) [minor isomer], 134.24 (s) [minor isomer], 134.55 (s) [major isomer], 161.00 (s) [mixed isomers], 161.45 (s) [minor isomer], 161.62 (s) [major isomer], 163.05 (s) [minor isomer], 163.34(s) [major isomer], 166.61 (s) [major isomer], 172.35 (s) [minor isomer], 191.36 (s) [mixed isomers] ppm.

HRMS Theoretical mass [M+H]+ 330.05485; Experimental mass [M+H]+ 330.05494

(E)-4-(((5-(2-hydroxyphenyl)-1,3,4-thiadiazol-2-yl)imino)methyl)benzene-1,3-diol (3)

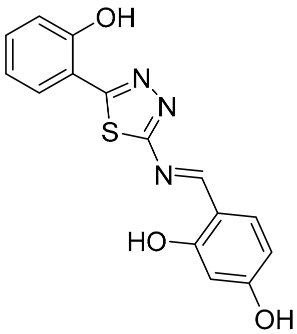

Yield: 51%; ^{1}H NMR (500 MHz, DMSO-d_6) δ = ppm 6.29 - 6.54 (m, 6 H)[mixed isomers], 7.49 - 7.57 (m, 1 H)[minor isomer], 7.68 (d, *J*=8.83 Hz, 1 H) [mixed isomers], 7.99 (d, *J*=8.83 Hz, 1 H) [major isomer], 9.07 (s, 1 H) [major isomer], 9.93 (s, 1 H) [minor isomer], 10.08 (s, 1 H) [major isomer], 10.66 (br. s., 1 H) [mixed isomers], 10.90 (s, 1 H) [minor isomer], 11.08 (br. s., 1 H) [major isomer], 11.94 (br. s., 1 H) [major isomer].

^{13}C NMR (126 MHz, DMSO-d6) δ = 102.64 (s) [major isomer], 102.87 (s) [mixed isomers], 102.99 (s) [minor isomer], 108.35 (s) [minor isomer], 108.81 (s) [mixed isomers], 109.08 (s) [mixed isomers], 109.14 (s) [mixed isomers], 109.56 (s) [mixed isomers], 112.59 (s) [mixed isomers], 115.68 (s), 129.07 (s) [major isomer], 129.20 (s) [minor isomer], 133.20 (s) [mixed isomers], 134.55 (s) [mixed isomers], 156.43 (s) [minor isomer], 156.78 (s) [major isomer], 160.22 (s) [minor isomer], 161.01 (s) [major isomer], 161.63 (s) [mixed isomers], 163.34 (s) [mixed isomers], 163.70 (s) [mixed isomers], 164.94 (s) [mixed isomers], 165.61 (s), 166.61 (s), 172.31 (s) [mixed isomers], 191.35 (s) [mixed isomers] ppm.

HRMS Theoretical mass [M+H]+ 314.05994; Experimental mass [M+H]+ 314.05981

(E)-4-(5-((4-(diethylamino)-2-hydroxybenzylidene)amino)-1,3,4-thiadiazol-2-yl)benzene-1,3-diol (4)

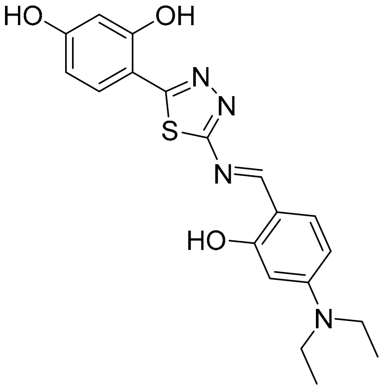

Yield: 42 %; ^{1}H NMR (500 MHz, DMSO-d6) δ ppm 1.07 - 1.20 (m, 6 H), 3.44 (q, *J*=7.04 Hz, 4 H), 6.15 (d, *J*=2.52 Hz, 1 H), 6.39 - 6.45 (m, 2 H), 6.46 - 6.51 (m, 1 H), 7.53 (d, *J*=9.14 Hz, 1 H), 7.95 (d, *J*=8.51 Hz, 1 H), 8.89 (s, 1 H), 10.01 - 10.08 (m, 1 H), 10.99 - 11.06 (m, 1 H), 12.11 - 12.36 (m, 1 H)

^{13}C NMR (126 MHz, DMSO-d6) δ = 12.76 (s), 44.64 (s), 79.65 (s), 96.54 - 97.50 (m), 102.6 (s), 105.62 (s), 108.76 (s), 108.85 (s), 109.25 (s), 129.12 (s), 153.66 (s), 156.70 (s), 160.21 (s), 161.44 (s), 163.65 (s), 166.01 (s), 172.45 (s) ppm

HRMS Theoretical mass [M+H]+ 385.13344; Experimental mass [M+H]+ 385.13349

(E)-4-(5-((4-(diethylamino)-2-hydroxybenzylidene)amino)-1,3,4-thiadiazol-2-yl)-2-methoxyphenol (5)

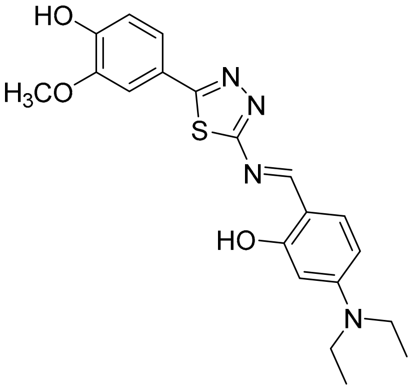

Yield: 36 %; ^{1}H NMR (500 MHz, DMSO-d6) δ = 1.12 - 1.16 (m, 6 H) [mixed isomers], 3.44 (q, *J*=6.83 Hz, 4 H) [mixed isomers], 3.87 (s, 3 H) [mixed isomers], 6.05 (d, *J*=2.21 Hz, 1 H) [minor isomer], 6.16 (d, *J*=2.21 Hz, 1 H) [major isomer], 6.33 - 6.36 (m, 1 H) [minor isomer], 6.43 (dd, *J*=8.99, 2.05 Hz, 1 H) [major isomer], 6.84 (d, *J*=8.20 Hz, 1 H) [minor isomer], 6.92 (d, *J*=8.20 Hz, 1 H) [major isomer], 7.09 (dd, *J*=8.20, 1.89 Hz, 1 H) [minor isomer], 7.24 - 7.27 (m, 1 H) [minor isomer], 7.33 (dd, *J*=8.20, 1.89 Hz, 1 H) [mixed isomers], 7.42 (d, *J*=8.83 Hz, 1 H) [minor isomer], 7.48 (d, *J*=2.21 Hz, 1 H) [major isomer], 7.55 (d, *J*=9.14 Hz, 1 H) [major isomer], 8.84 (s, 1 H) [major isomer], 9.50 - 9.54 (m, 1 H) [minor isomer], 9.61 (s, 1 H) [minor isomer], 9.82 (s, 1 H) [major isomer], 11.22 - 11.28 (m, 1 H) [minor isomer], 11.80 - 12.12 (m, 1 H) [major isomer] ppm.

^{13}C NMR (126 MHz, DMSO-d6) δ = 12.90 (s) [minor isomer], 13.02 (s) [major isomer], 44.59 (s) [minor isomer], 44.69 (s) [major isomer], 56.08 (s) [minor isomer], 56.16 (s) [major isomer], 96.40 (s) [minor isomer], 96.95 (s) [major isomer], 104.94 (s) [minor isomer], 105.94 (s) [major isomer], 108.88 (s) [mixed isomers], 109.78 (s) [mixed isomers], 110.51 (s) [mixed isomers], 111.70 (s) [mixed isomers], 116.17 (s) [minor isomer], 116.47 (s) [major isomer], 121.63 (s) [major isomer], 122.05 (s) [minor isomer], 148.56 (s) [major isomer], 150.09 (s) [minor isomer], 153.99 (s) [mixed isomers], 163.70 (s) [minor isomer], 164.44 (s) [major isomer] ppm.

HRMS Theoretical mass [M+H]+ 399.14909; Experimental mass [M+H]+ 399.14902

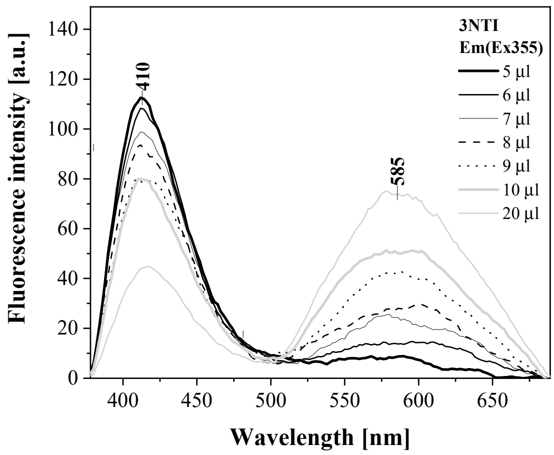

Figure S1. Fluorescence emission spectra for 3NTI at concentrations corresponding to the biological results, at the wavelength corresponding to the main absorption spectrum maximum.

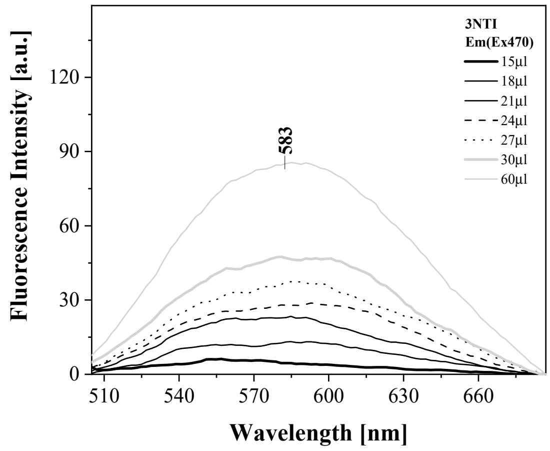

Figure S2. Fluorescence emission spectra for 3NTI at concentrations corresponding to the biological results, at the wavelength corresponding to aggregated structures.

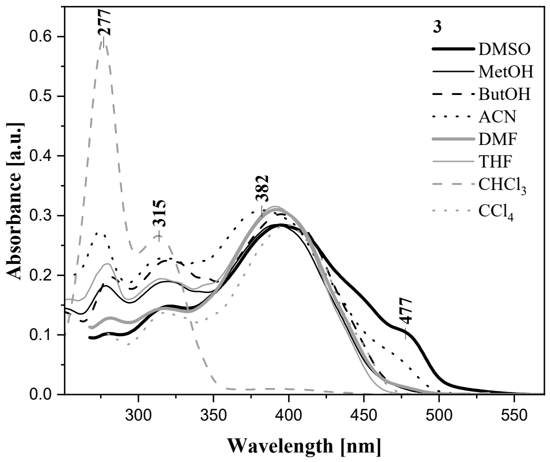

Figure S3. Electronic absorption spectra for analogue no. 3 in selected solvents of varying polarity.

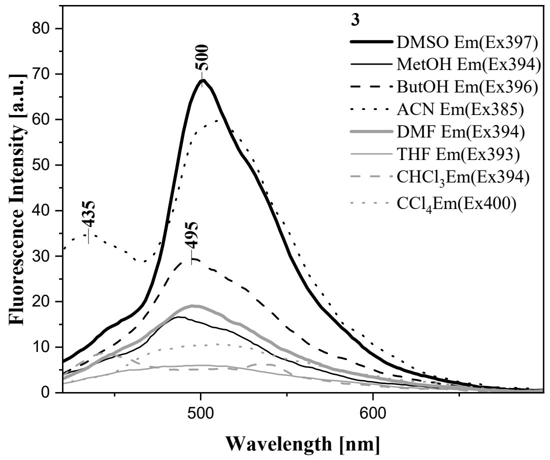

Figure S4. Fluorescence emission spectra for analogue no. 3, corresponding to the spectra shown in Figure S1, at a greater excitation wavelength.

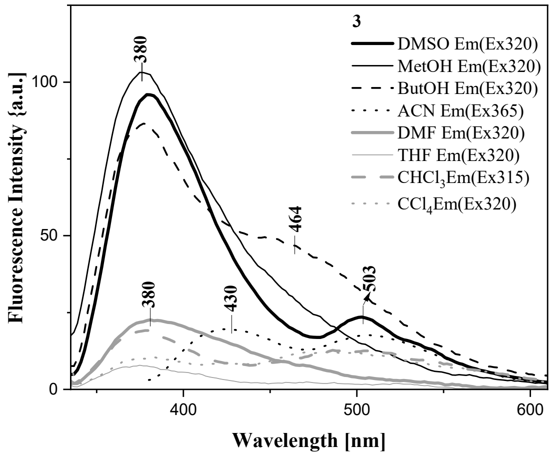

Figure S5. Fluorescence emission spectra for analogue no. 3, corresponding to the spectra shown in Figure S1, at a shorter excitation wavelength.

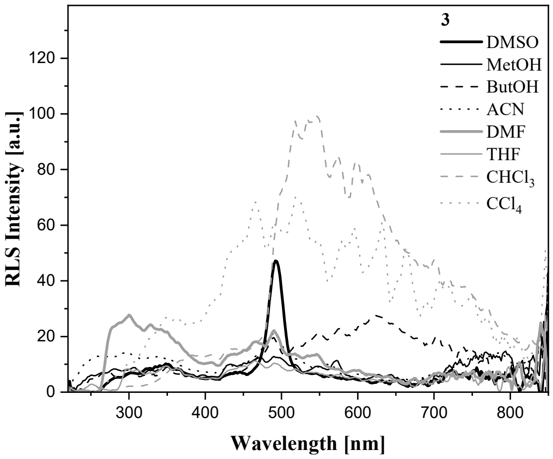

Figure S6. Resonance light scattering spectra for analogue no. 3, corresponding to the spectra Show in Figure S1.

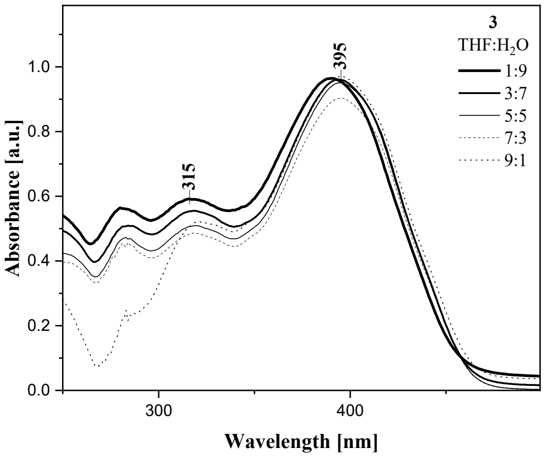

Figure S7. Electronic absorption spectra for analogue no. 3, in the solvent mixture THF:H_2O.

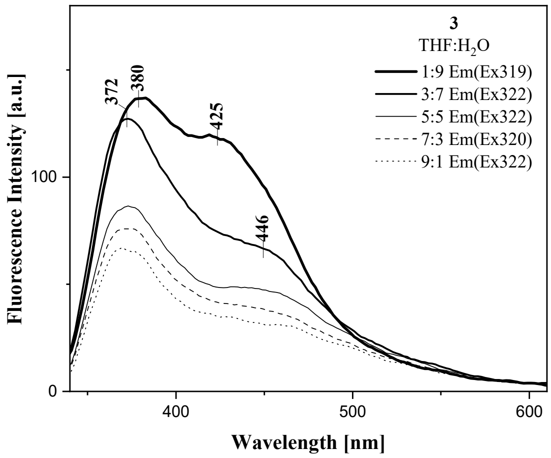

Figure S8. Fluorescence emission spectra for analogue no. 3 in the solvent mixture THF:H_2O, corresponding to those shown in Figure S5.

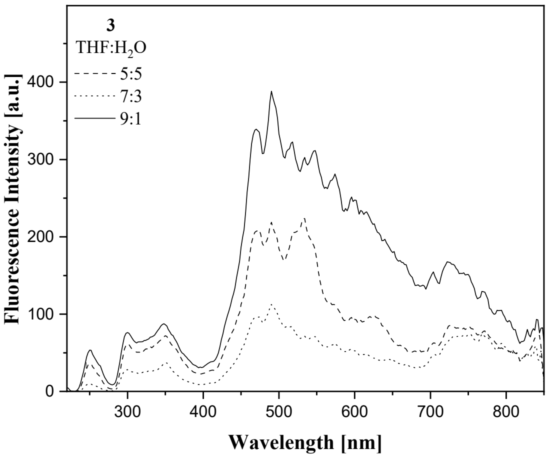

Figure S9. RLS spectra for analogue no. 3 in the solvent mixture THF:H_2O, corresponding to those shown in Figure S5.

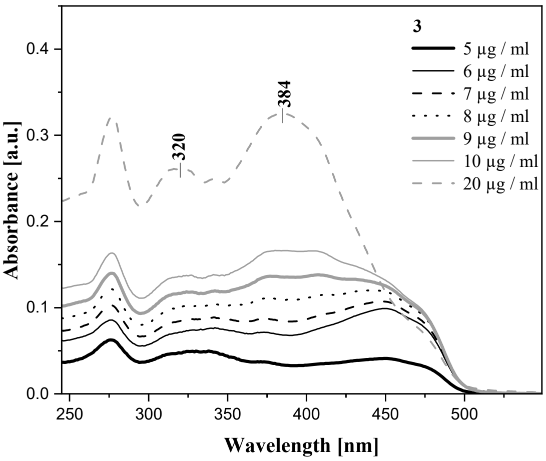

Figure S10. Electronic absorption spectra for analogue no. 3 at concentrations correspodning to the biological results.

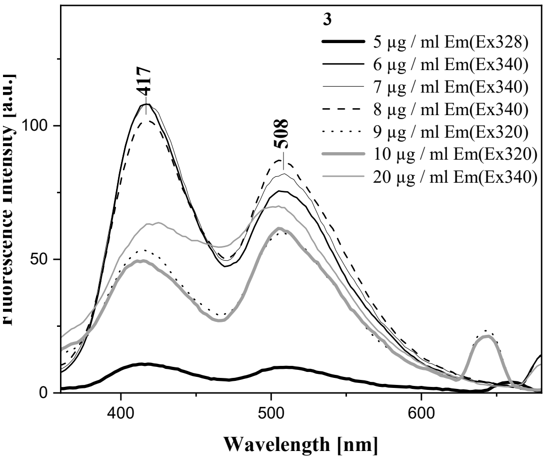

Figure S11. Fluorescence emission spectra for analogue no. 3 at concentrations corresponding to the biological results.

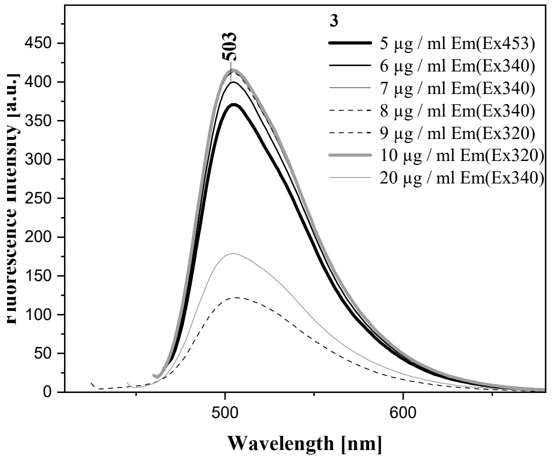

Figure S12. Fluorescence emission spectra for analogue no. 3 at concentrations corresponding to the biological results, at wavelengths corresponding to aggregated structures.

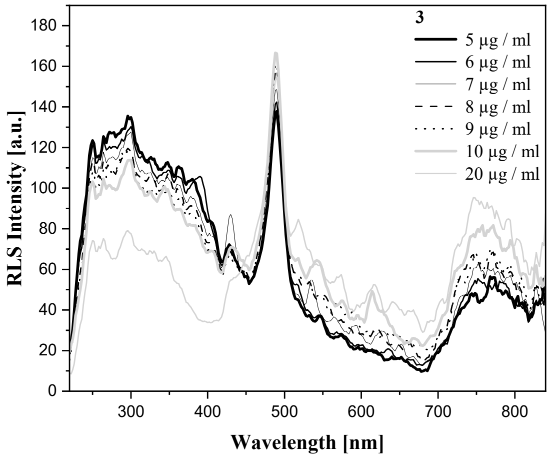

Figure S13. RLS spectra for analogue no. 3 at concentrations corresponding to the biological results.

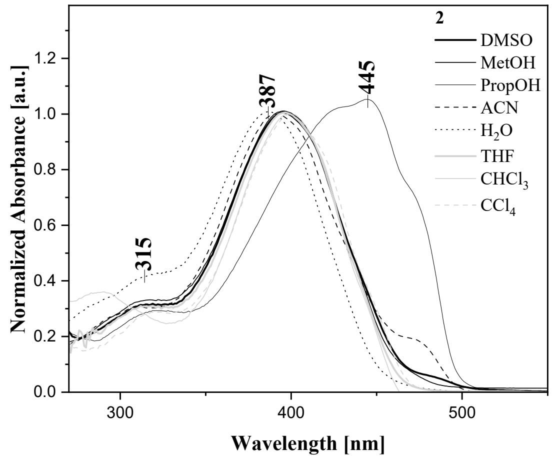

Figure S14. Electronic absorption spectra for analogue no. 2 in the several chosen solvents of different polarity

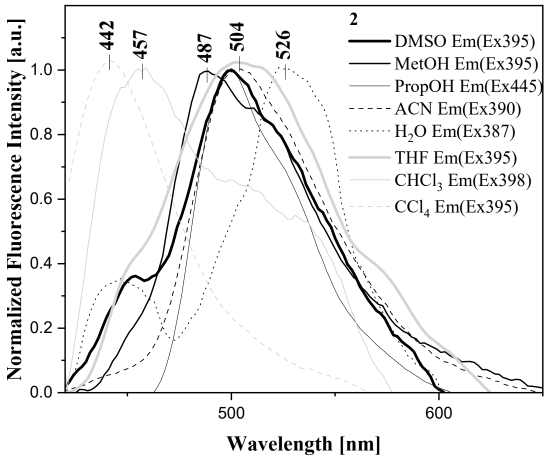

Figure S15. Fluorescence emission spectra for analogue no. 2 corresponding to electron emission spectra from Figure S14.

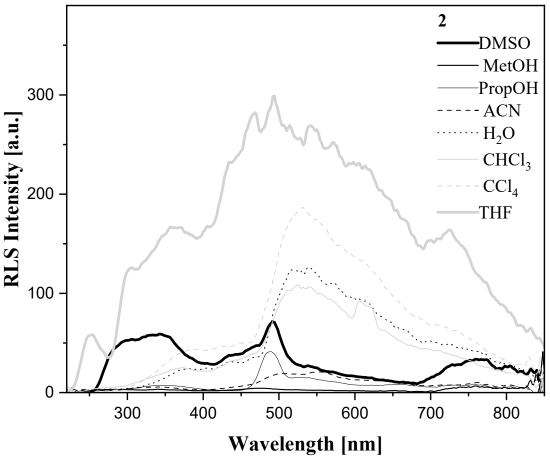

Figure S16. RLS spectra for analogue no. 2

Table S2. Examples of fluorescence lifetime measurements for analogue **3** in the selected solvents.

Solvent	τ_{av}	τ_1 [ns]	share	τ_2 [ns]	share	τ_3 [ns]	share
ACN	0.267	1.001	17.64%	0.06579	33.83	0.14137	48.53
Butanol	0.182	1.006	1.95	0.02136	36.73	0.25238	61.32
CCl4	0.408	3.474	10.94	0.01705	85.96	0.4219	3.10
CHCl3	0.766	2.347	31.25	0.02409	62.54	0.2878	6.20
DMF	1.191	0.374	41.00	0.0826	29.13	3.3913	29.87
DMSO	0.3360	2.347	4.20	0.2681	83.38	0.1119	12.24
MtOH	0.147	2.340	3.94	0.0363	69.53	0.112	26.53
THF	0.141	1.334	7.61	0.0110	80.23	0.252	12.17
DMSO	0.1419	0.8274	4.79	0.1239	70.67	0.06003	24.54
ACN	0.2534	0.2849	77.48	0.1708	17.73	0.05572	4.79

Table S3. Examples of fluorescence lifetime for analogue 3 in the solvent mixture THF:H_2O.

THF:H2O	τ_{av}	τ_1 [ns]	share	τ_2 [ns]	share	τ_3 [ns]	share
1:9	0.6516	2.5725	21.38	0.03895	49.03	0.2786	29.59
3:7	0.140	1.681	3.38	0.02889	54.56	0.1595	42.07

5:5	0.121	0.145	34.89	0.01931	43.18	0.2838	21.93
7:3	0.177	0.305	31.31	0.02219	25.87	0.1767	42.82
9:1	0.216	0.307	47.75	0.01721	17.17	0.1907	35.07

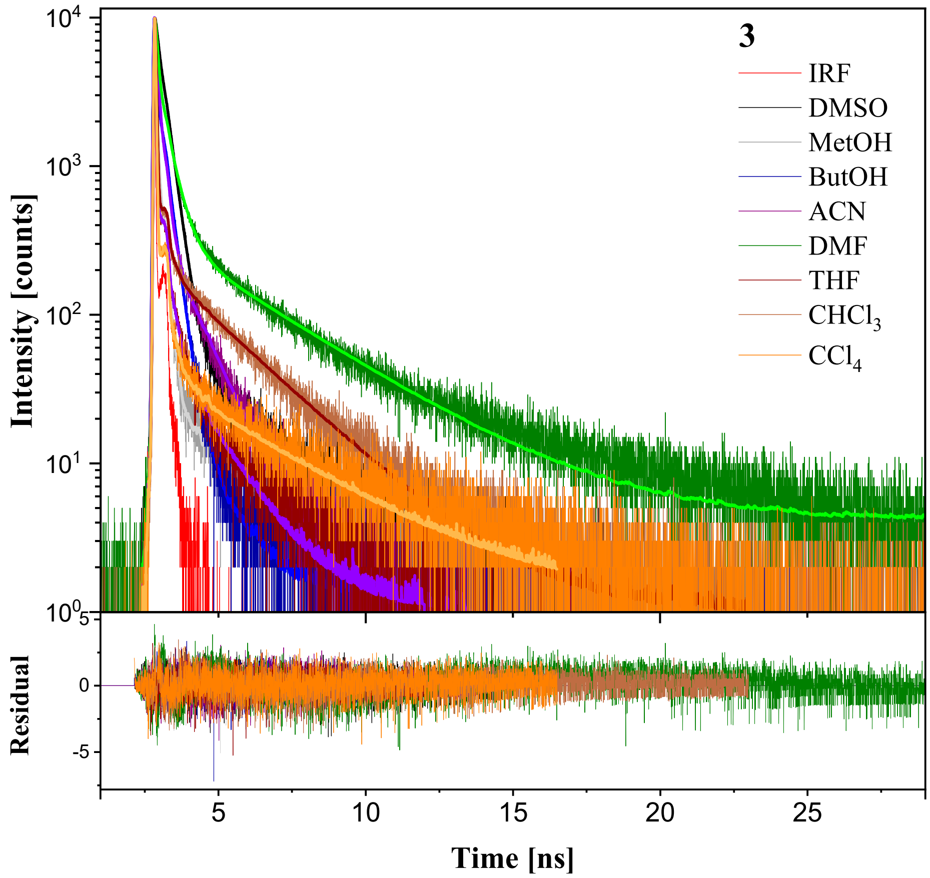

Figure S17. Example fluorescence lifetime curves in selected solvents, for analogue no. 3.

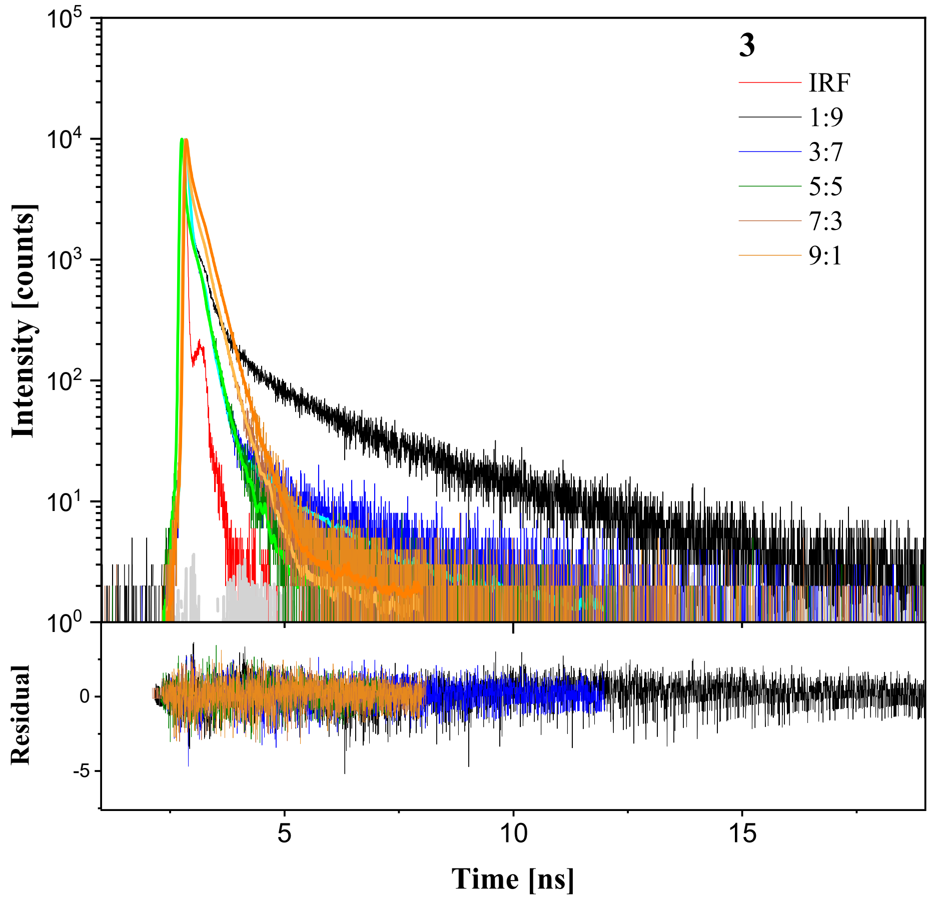

Figure S18. Example fluorescence lifetime curves for analogue no. 3, in solvent mixture THF:H_2O.

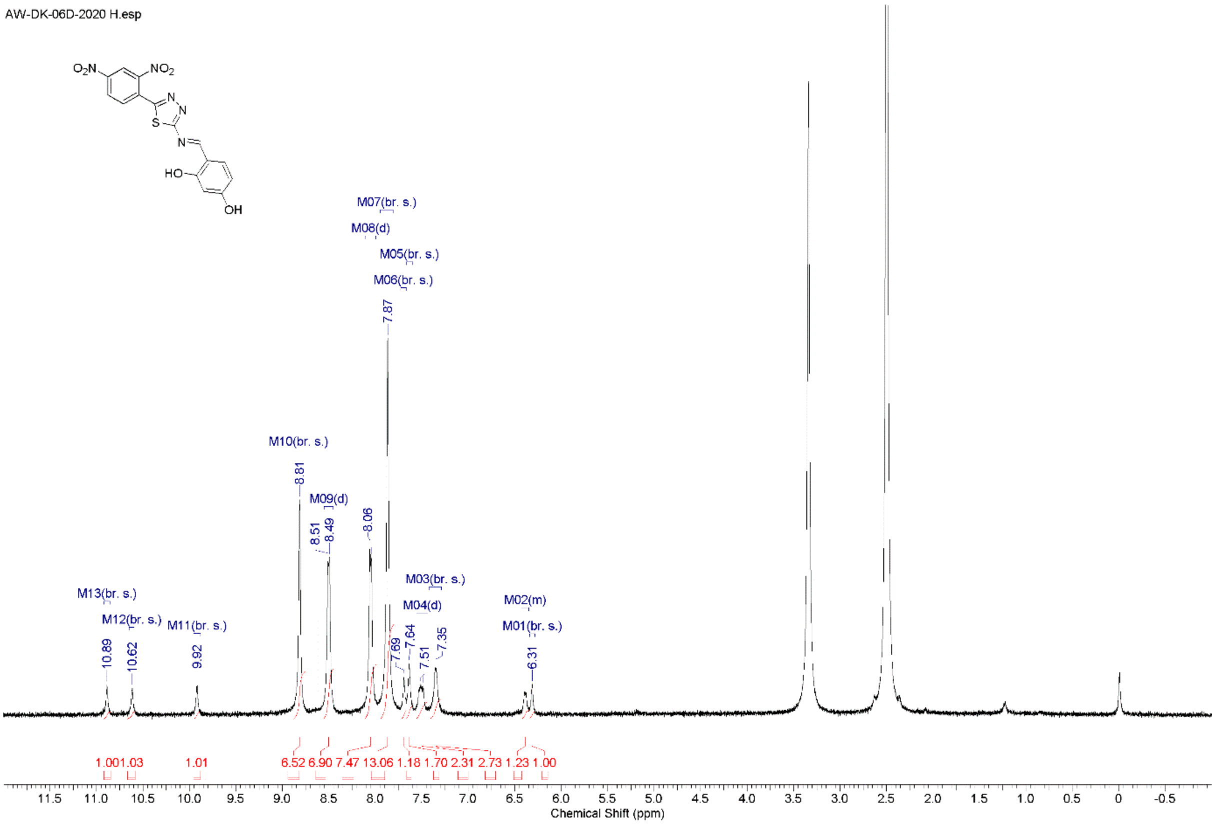

Figure S19. ^{1}H NMR Spectra of 3NTI

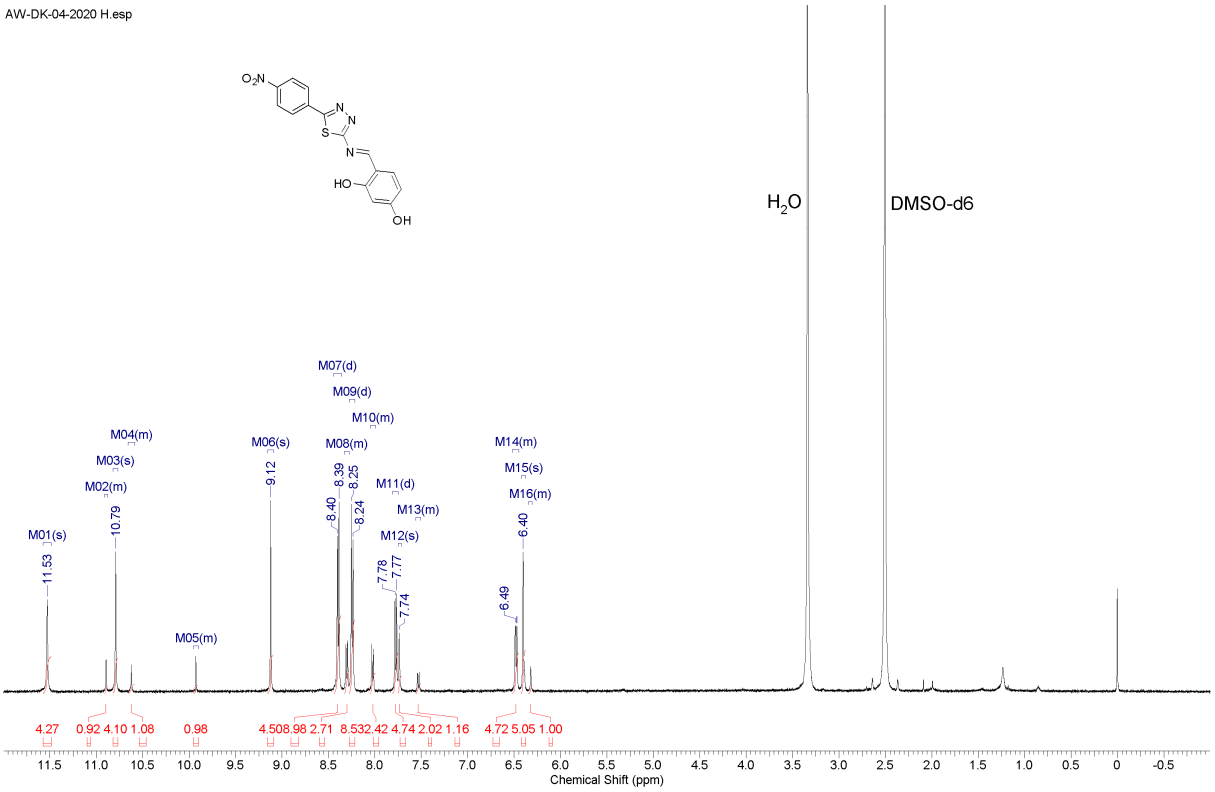

Figure S20. ^{1}H NMR Spectra of 1

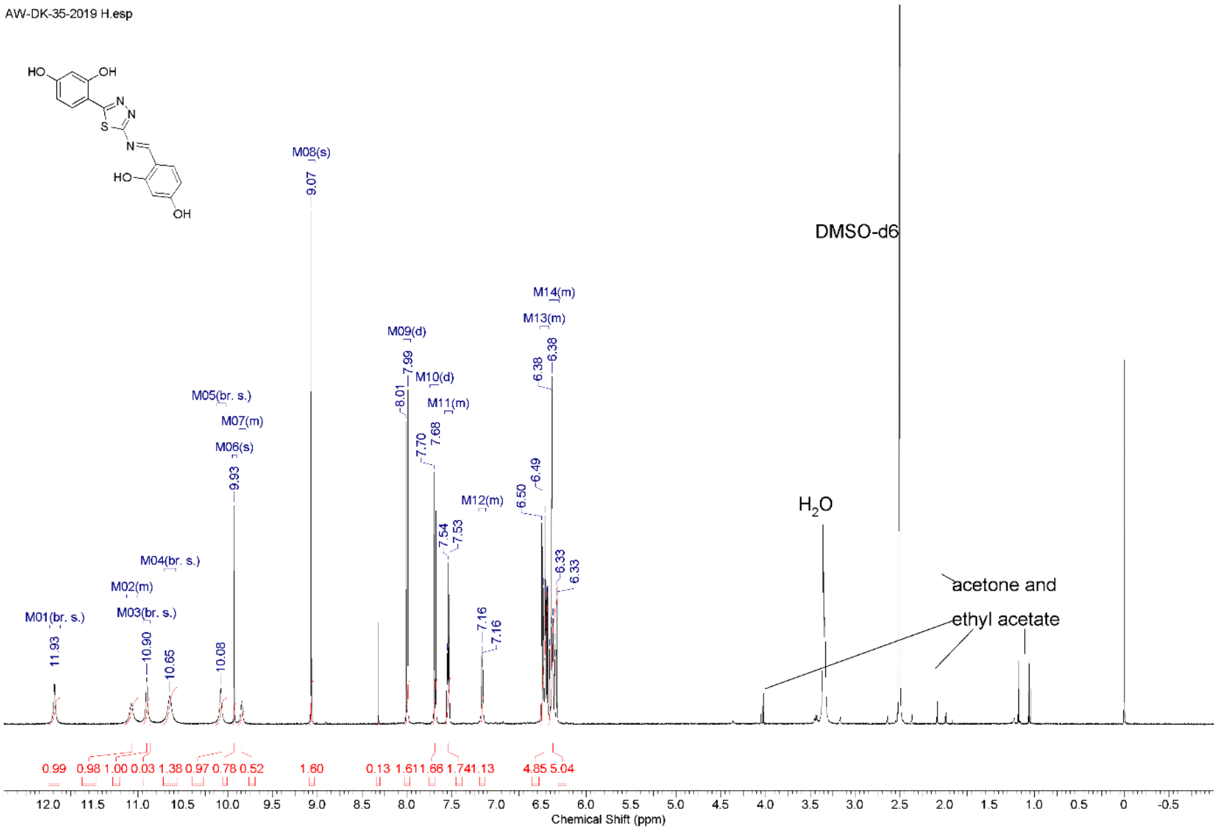

Figure S21. ^{1}H NMR Spectra of 2

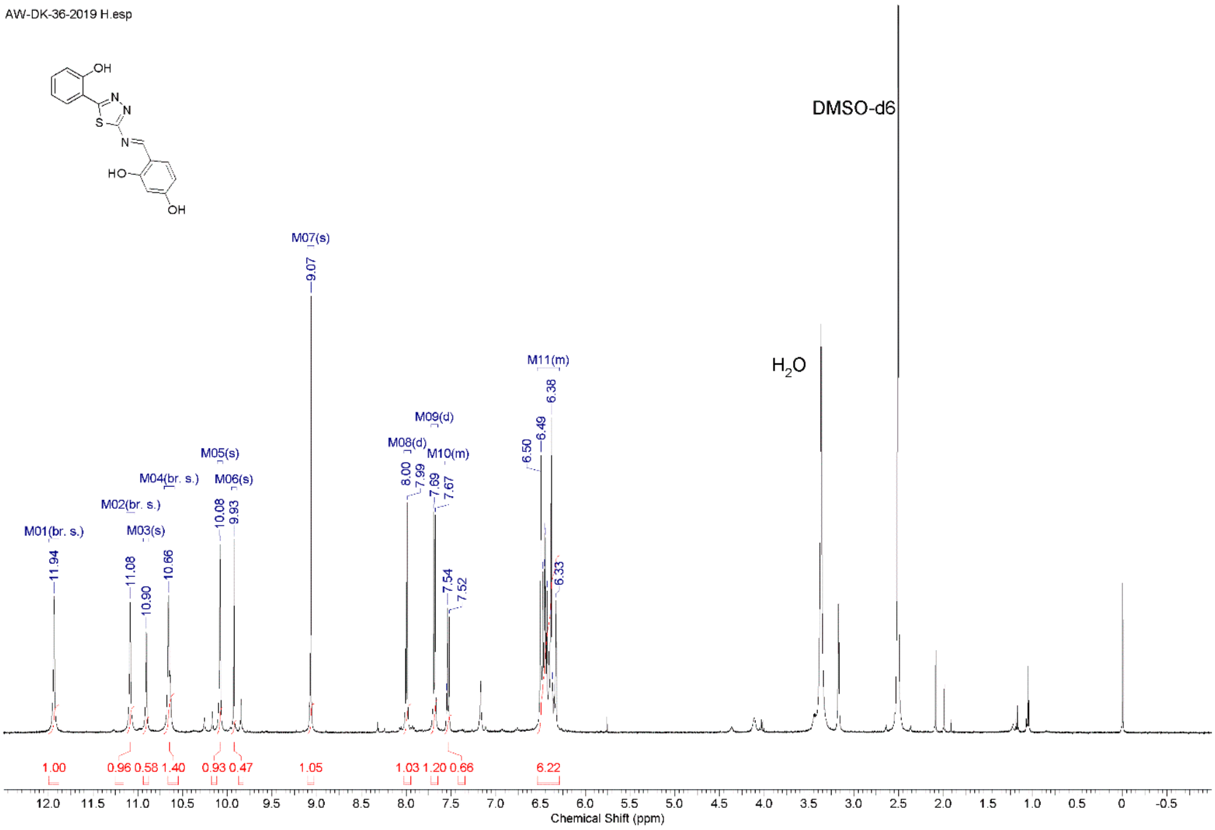

Figure S22. ^{1}H NMR Spectra of 3

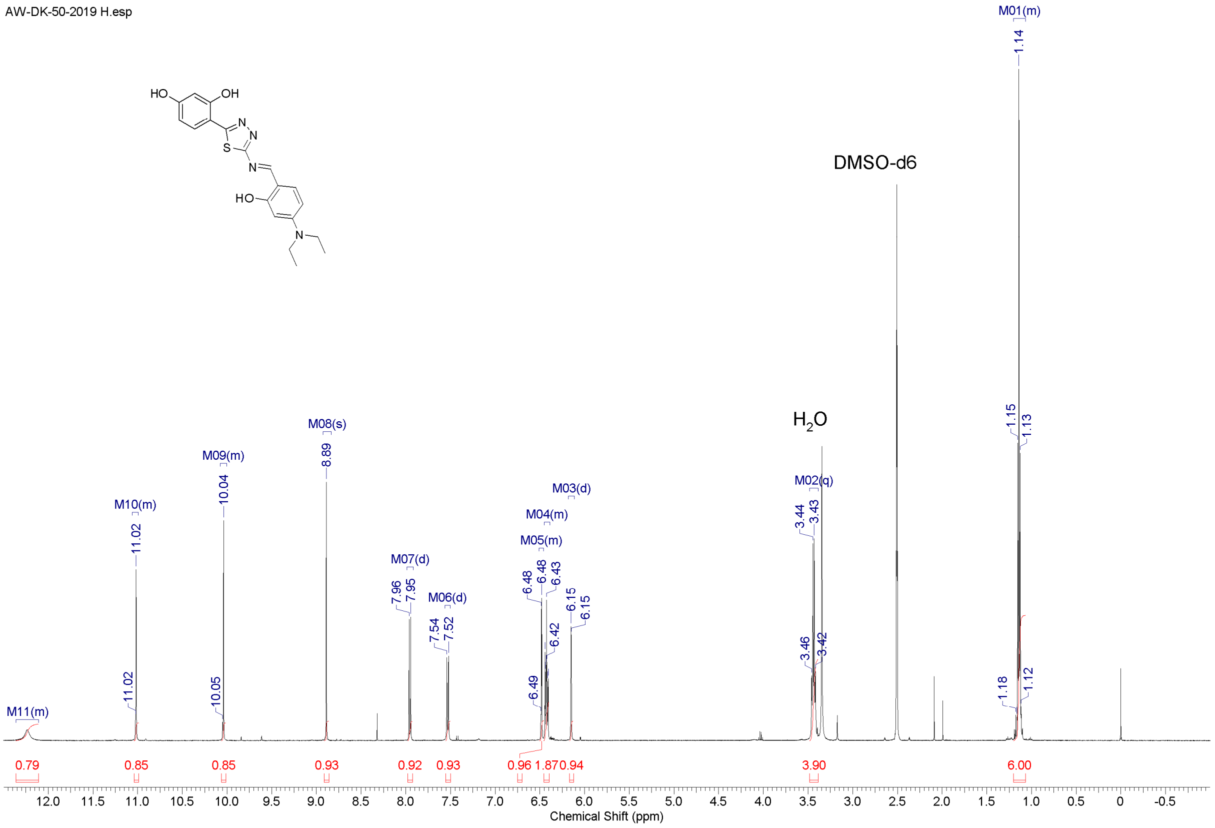

Figure S23. ^{1}H NMR Spectra of 4

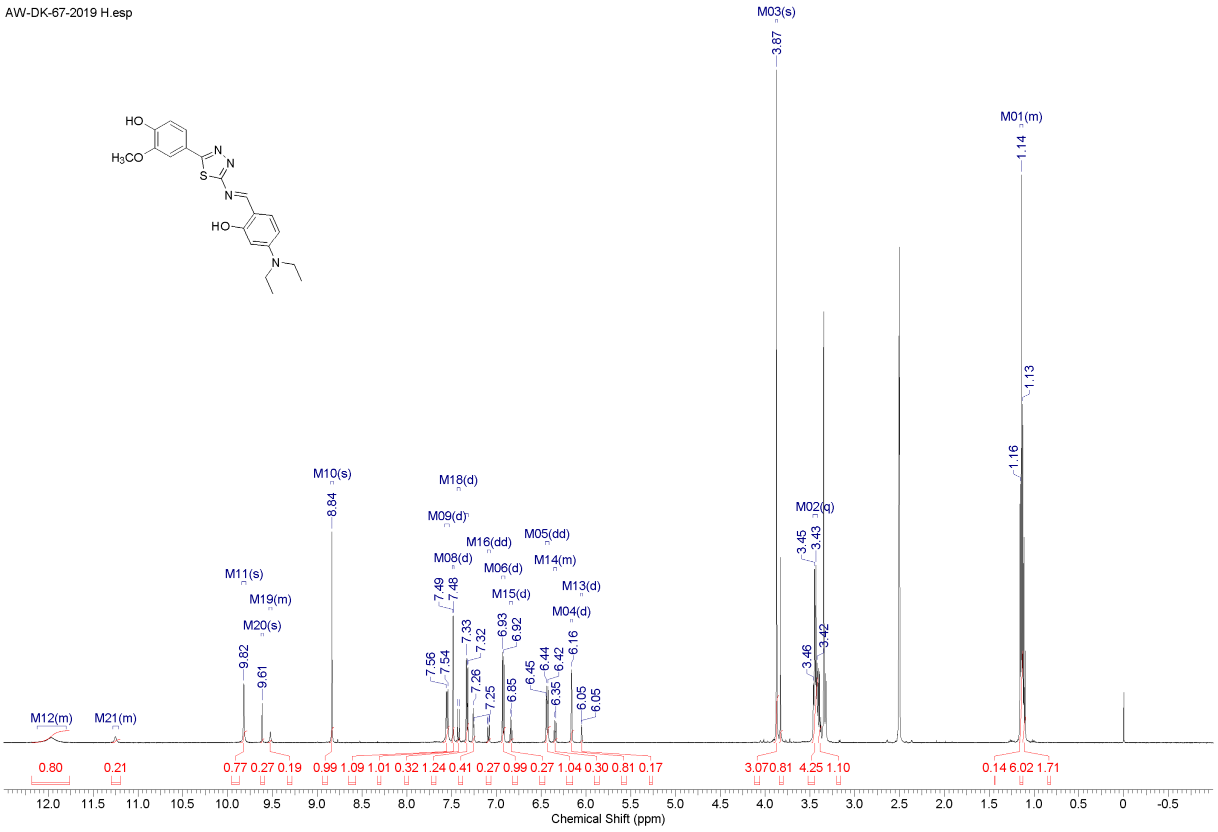

Figure S24. ¹H NMR Spectra of 5

Single crystal x-ray diffraction of **(E)-4-(5-((4-(diethylamino)-2-hydroxybenzylidene)amino)-1,3,4-thiadiazol-2-yl)-2-methoxyphenol (5)**

The structure of (E)-4-(5-((4-(diethylamino)-2-hydroxybenzylidene)amino)-1,3,4-thiadiazol-2-yl)-2-methoxyphenol was confirmed by single crystal x-ray diffraction were measured at room temperature on a SuperNova diffractometer with CuKα radiation (λ = 1.54184 Å). The CrysAlisPro (2014, Oxfordshire, UK program was used for data collection, cell refinement and data reduction with all necessary corrections. The structure was solved by direct methods using SHELXT[2] and refined by a least-squares method on F^2 using the SHELXL[3]. All non-hydrogen atoms were refined with anisotropic atomic displacement parameters while hydrogen atoms were positioned geometrically with isotropic displacements. The structure is deposited in the Cambridge Crystallographic Data [redacted] Centre under number #2417699 [117,118].

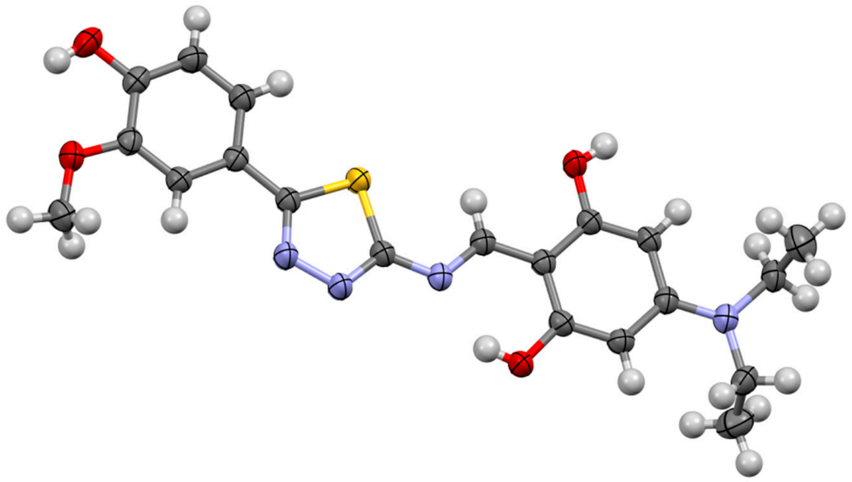

Figure S25. Molecular structures of (E)-4-(5-((4-(diethylamino)-2-hydroxybenzylidene)amino)-1,3,4-thiadiazol-2-yl)-2-methoxyphenol observed in the solid. The thermal ellipsoids are drawn at a 50% probability level.

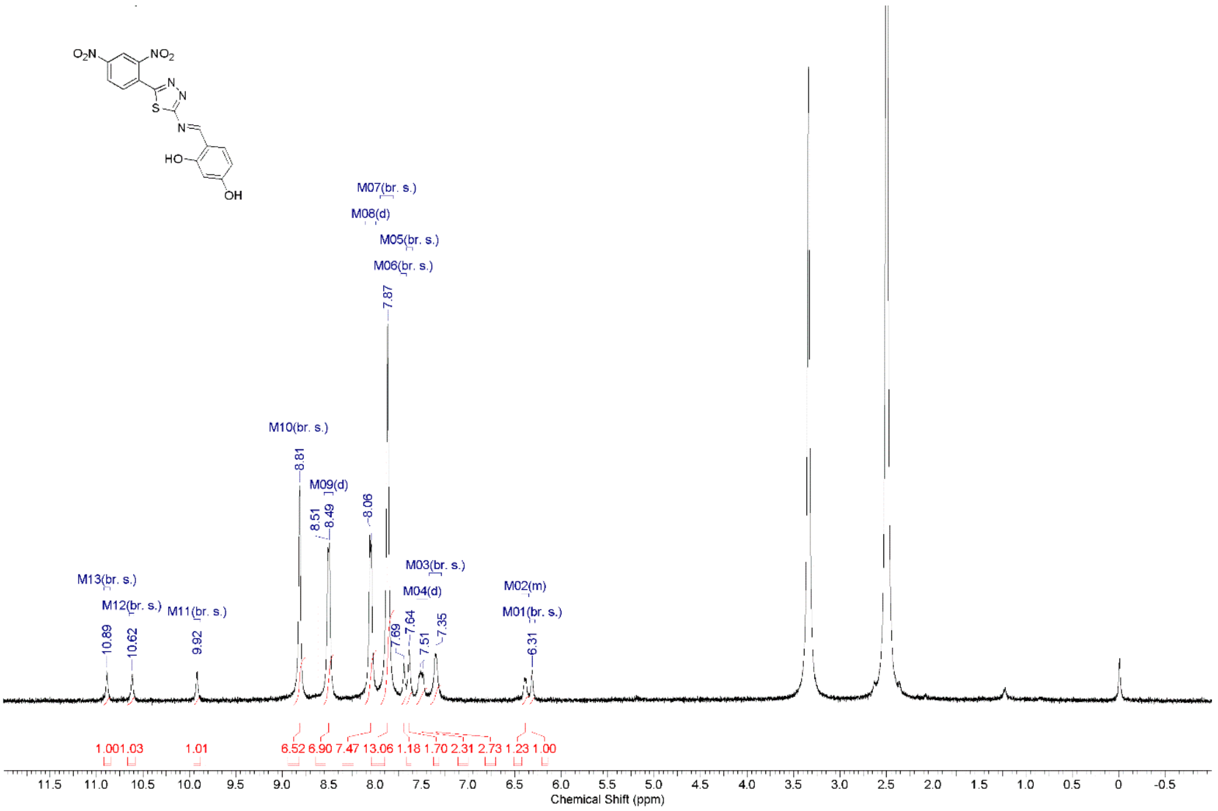

Figure S26. ^{1}H NMR Spectra of 3NTI.

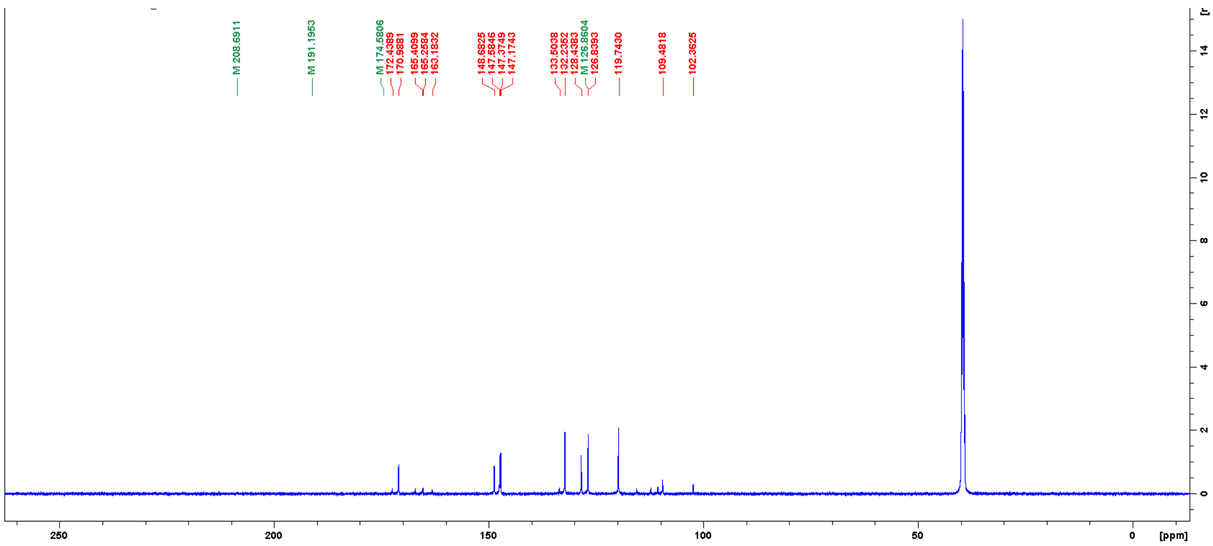

Figure S27. ^{13}C NMR Spectra of 3NTI.

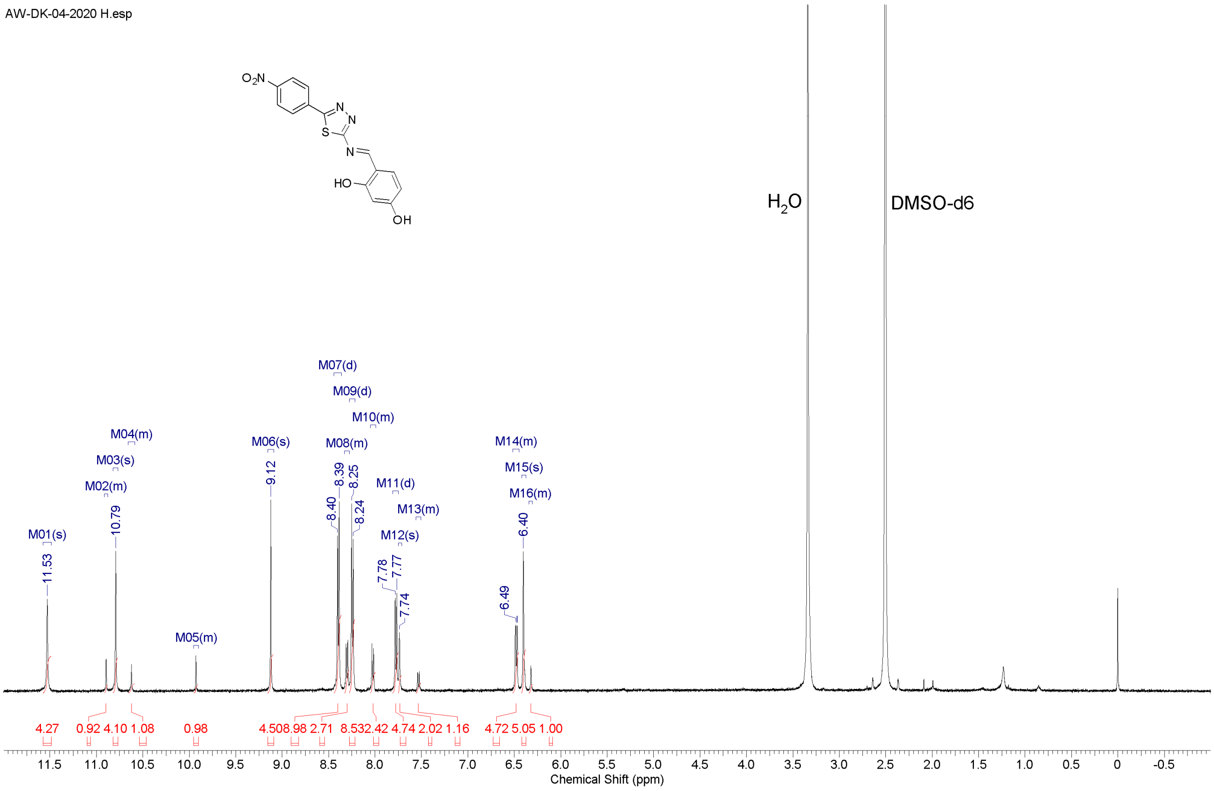

Figure S28. ^{1}H NMR Spectra of 1.

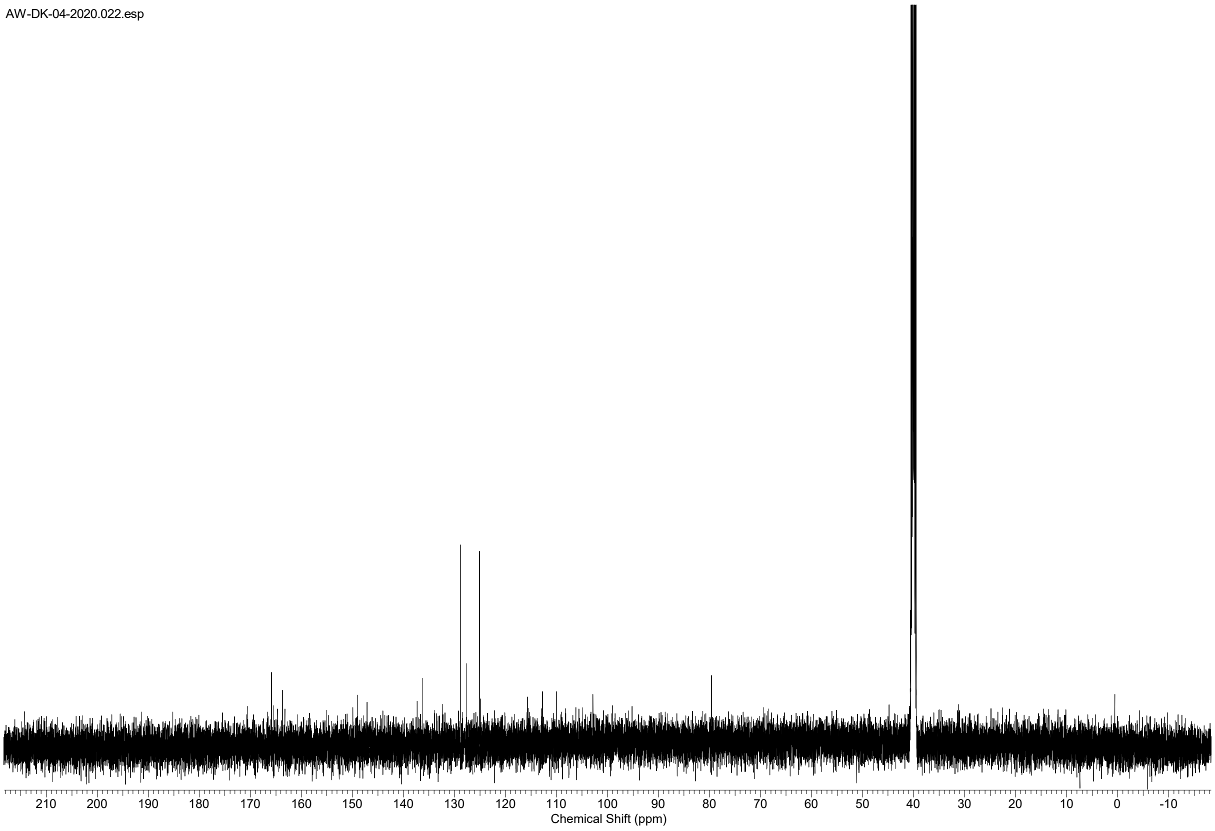

Figure S29. ^{13}C NMR Spectra of 1.

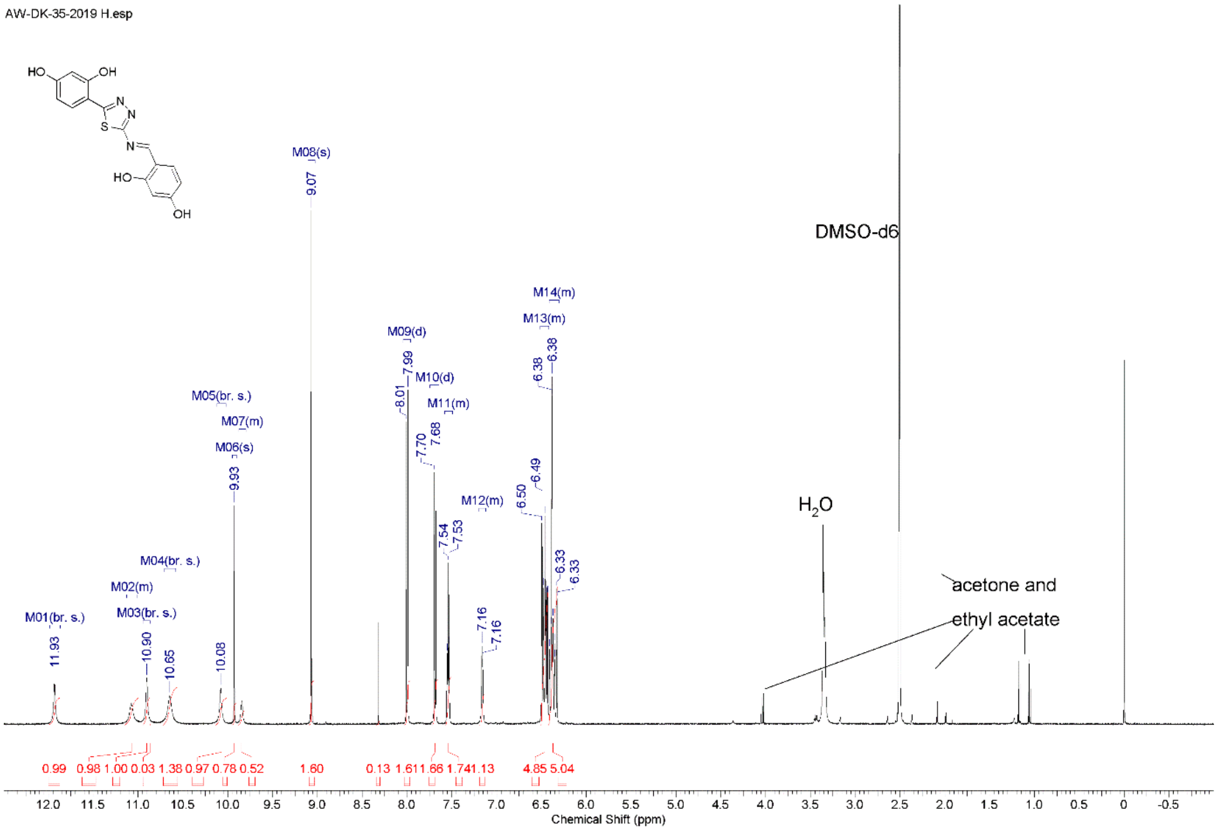

Figure S30. ^{1}H NMR Spectra of 2.

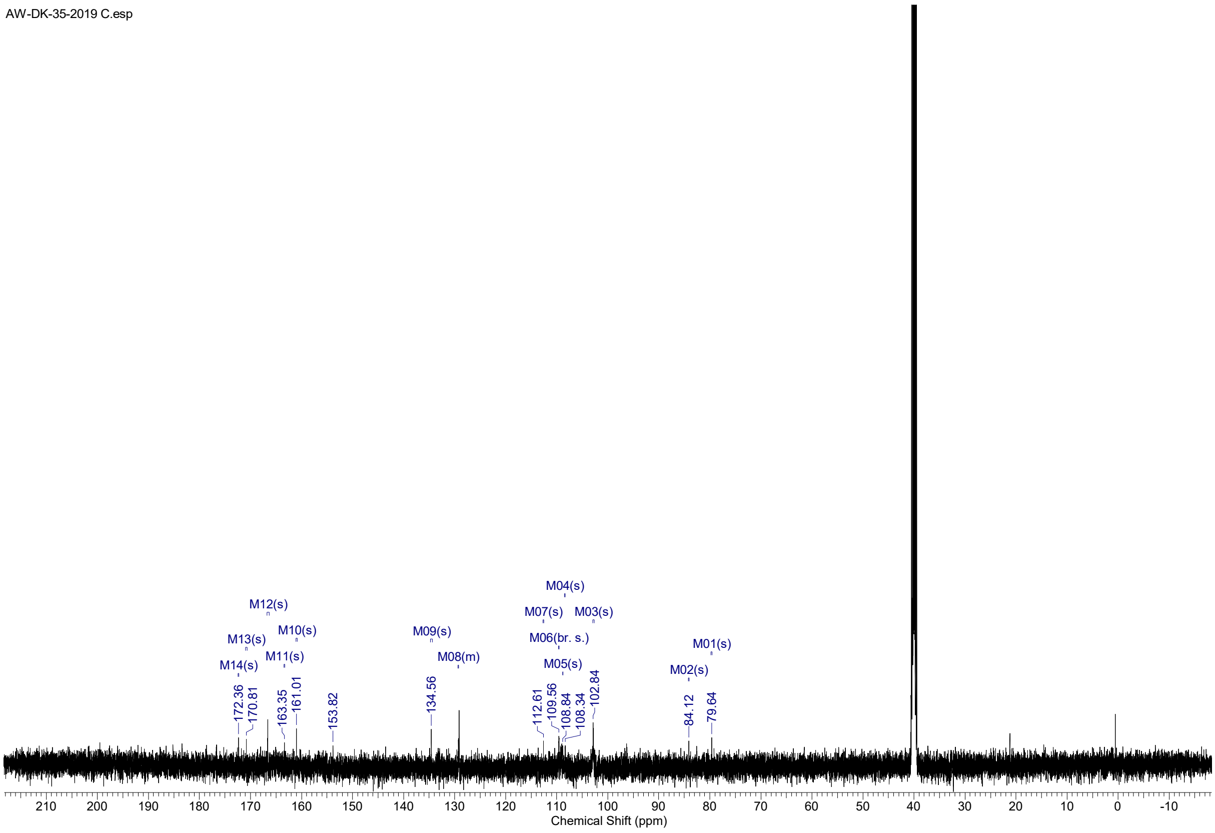

Figure S31. ^{13}C NMR Spectra of 2.

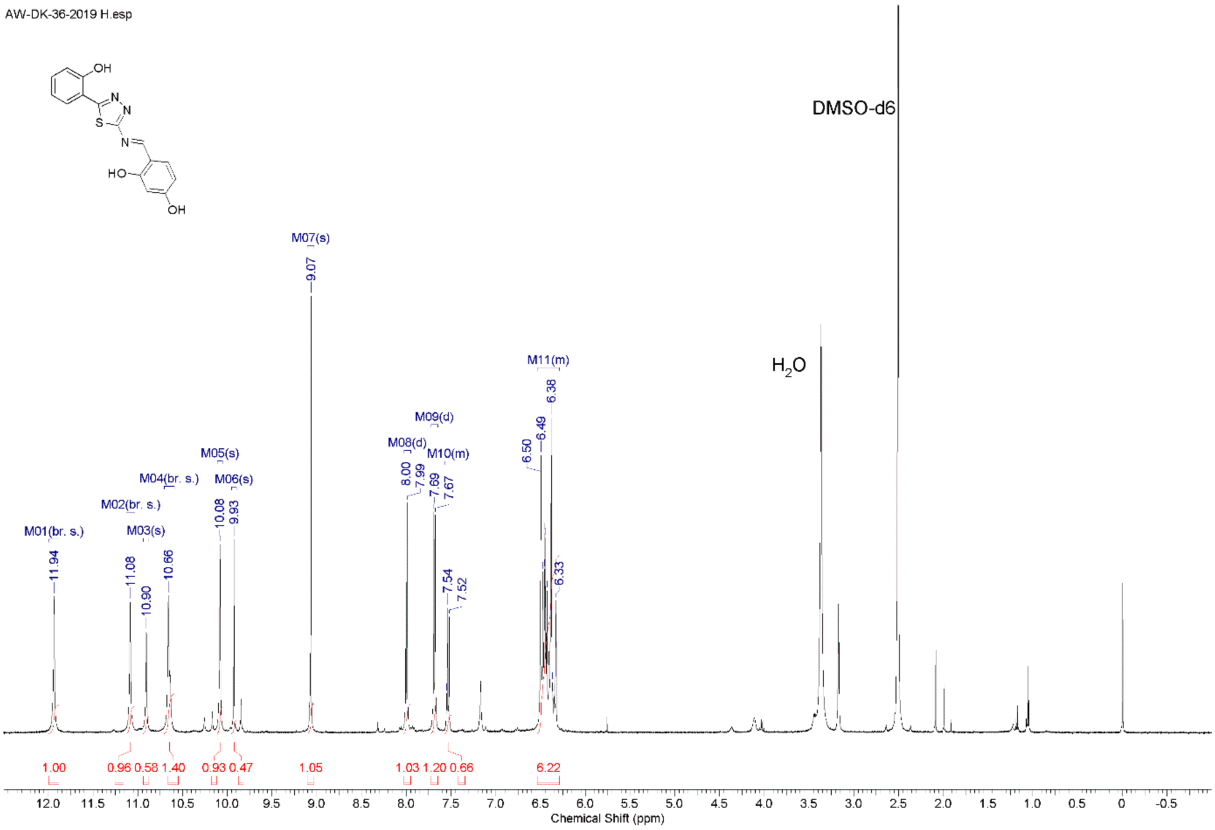

Figure S32. ^{1}H NMR Spectra of 3.

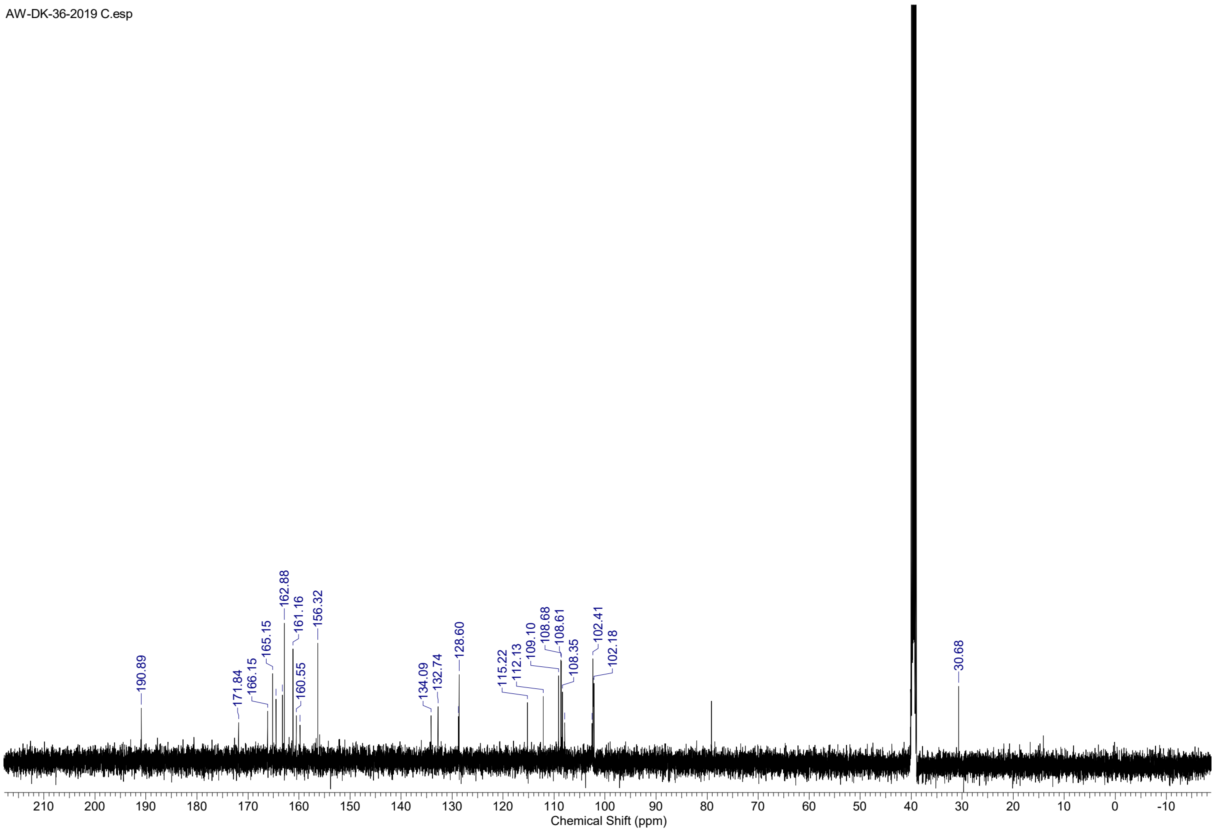

Figure S33. ^{13}C NMR Spectra of 3.

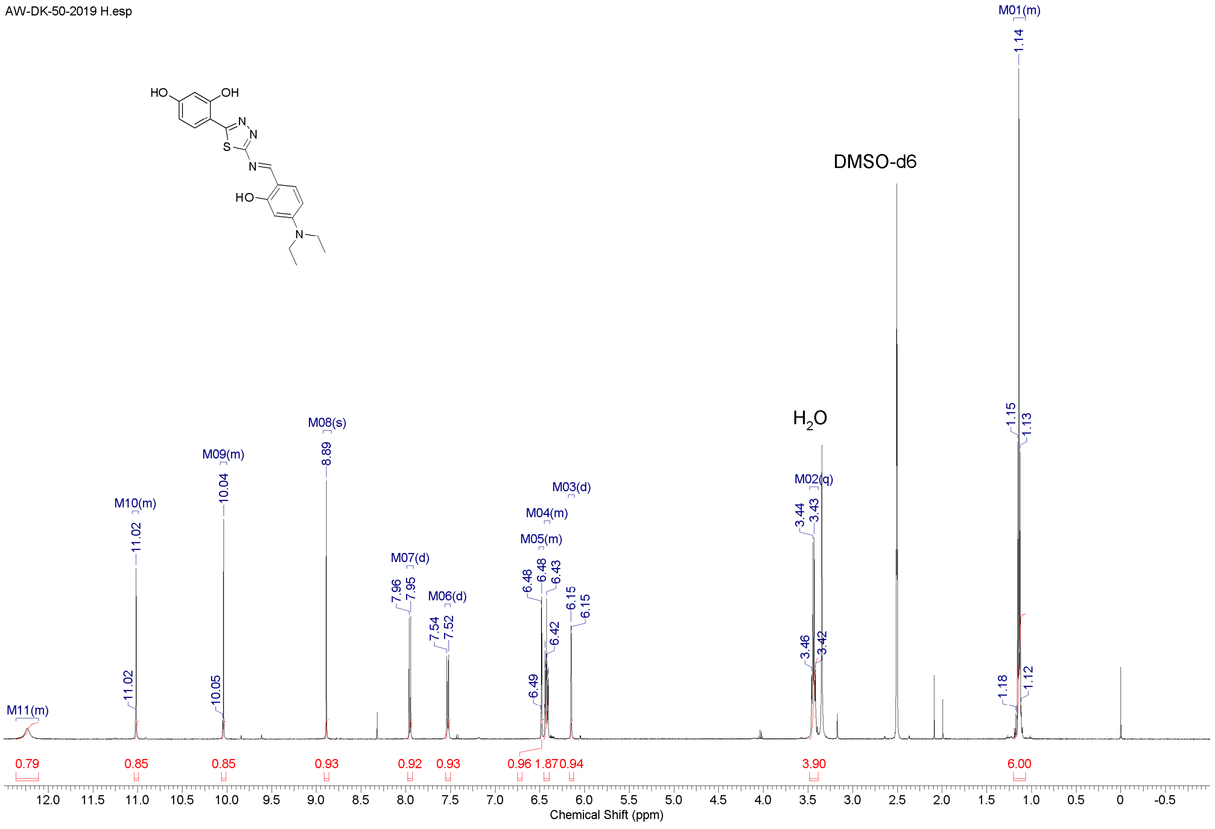

Figure S34. ^{1}H NMR Spectra of 4.

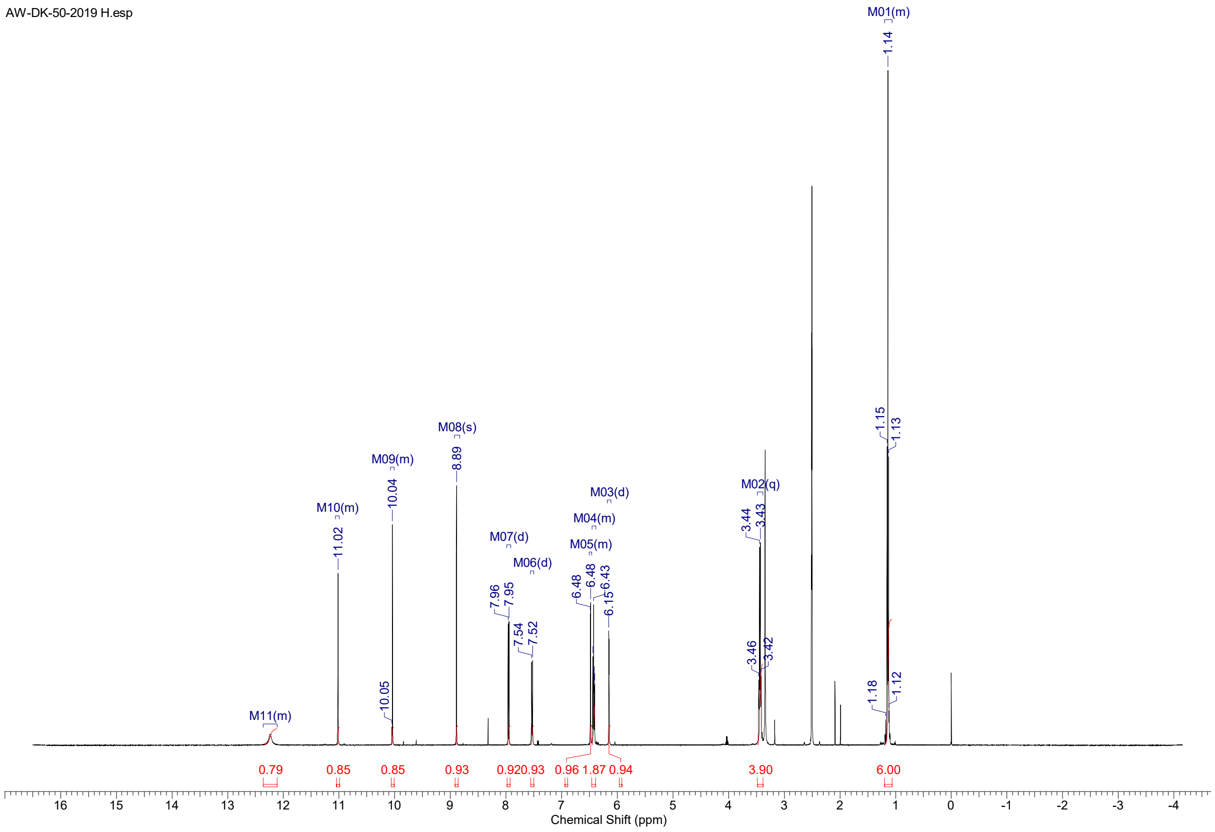

Figure S35. ^{13}C NMR Spectra of 4.

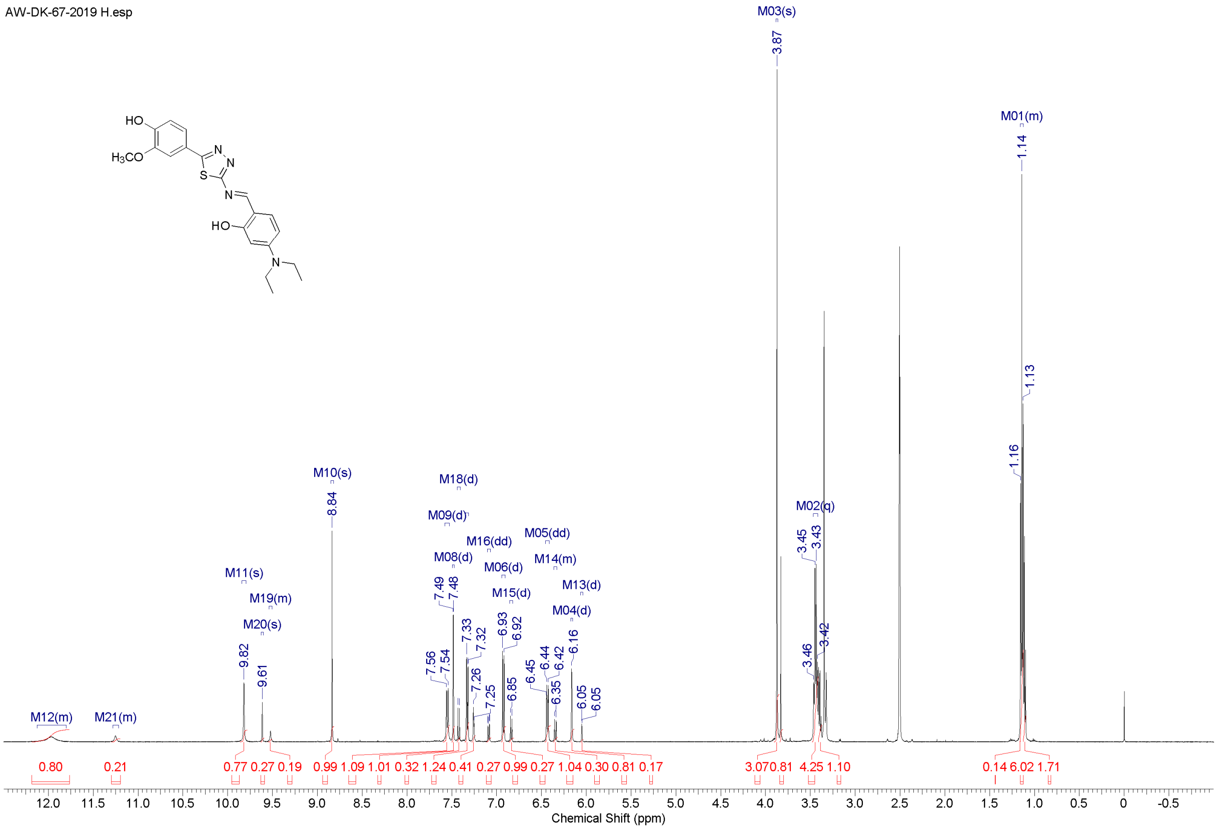

Figure S36. ^{1}H NMR Spectra of 5.

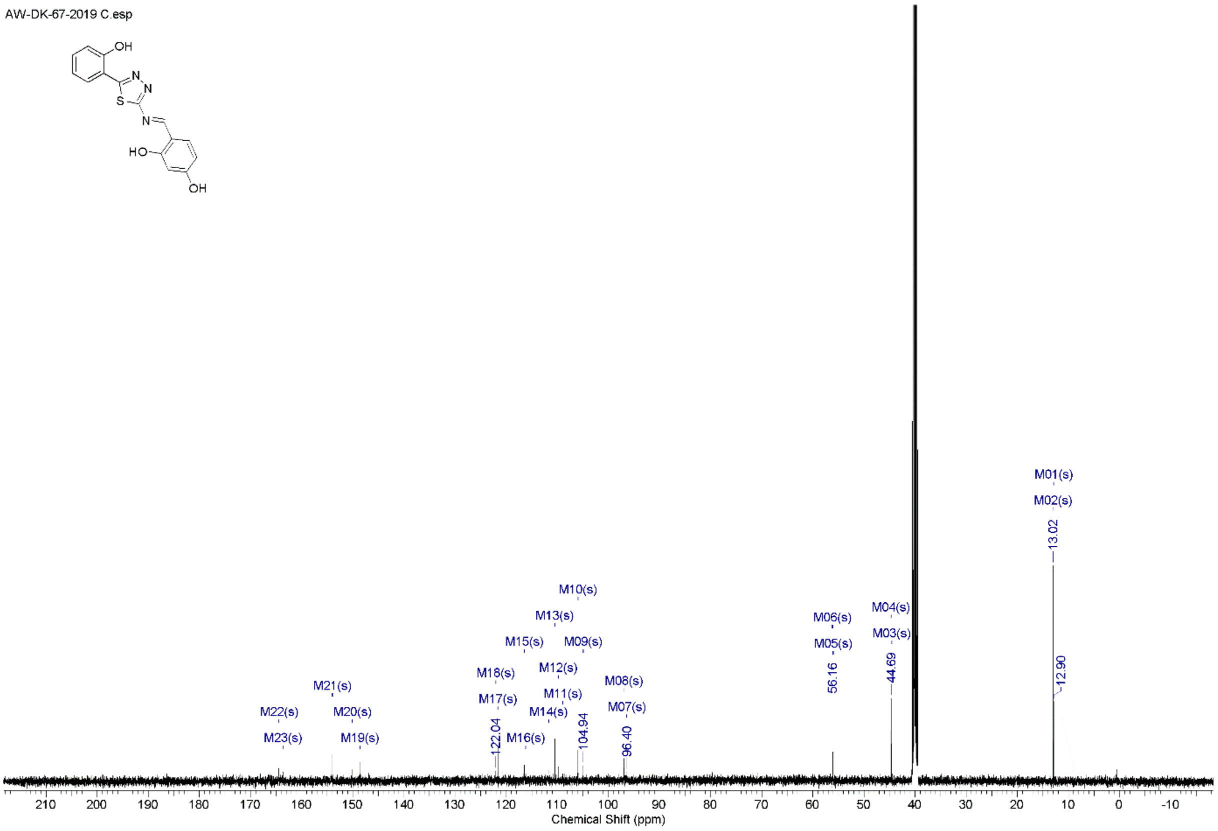

Figure S37. ^{13}C NMR Spectra of 5.

1. Sheldrick, G.M. SHELXT–Integrated Space-Group and Crystal-Structure Determination. *Acta Crystallographica Section A: Foundations and Advances* **2015**, *71*, 3–8.
2. Sheldrick, G.M. Crystal Structure Refinement with SHELXL. *Acta Crystallographica Section C: Structural Chemistry* **2015**, *71*, 3–8.
